# New Insights into Samango Monkey Speciation in South Africa

**DOI:** 10.1371/journal.pone.0117003

**Published:** 2015-03-23

**Authors:** Desiré L. Dalton, Birthe Linden, Kirsten Wimberger, Lisa Jane Nupen, Adrian S. W. Tordiffe, Peter John Taylor, M. Thabang Madisha, Antoinette Kotze

**Affiliations:** 1 National Zoological Gardens of South Africa, Pretoria, South Africa; 2 Genetics Department, University of the Free State, Bloemfontein, South Africa; 3 Department of Zoology, School of Mathematical & Natural Sciences, University of Venda, Thohoyandou, South Africa; 4 SARChI Chair on Biodiversity Value & Change in the Vhembe Biosphere Reserve & Core Member of Centre for Invasion Biology, School of Mathematical & Natural Sciences, University of Venda, Thohoyandou, South Africa; 5 Department of Biological Sciences, University of Cape Town, Rondebosch, South Africa; 6 Percy FitzPatrick Institute, Department of Biological Sciences, University of Cape Town, Rondebosch, South Africa; 7 Department of Companion Animal Clinical Studies, Faculty of Veterinary Science, University of Pretoria, Onderstepoort, South Africa; Centers for Disease Control and Prevention, UNITED STATES

## Abstract

The samango monkey is South Africa's only exclusively forest dwelling primate and represents the southernmost extent of the range of arboreal guenons in Africa. The main threats to South Africa's forests and thus to the samango are linked to increasing land-use pressure and increasing demands for forest resources, resulting in deforestation, degradation and further fragmentation of irreplaceable habitats. The species belongs to the highly polytypic *Cercopithecus nictitans* group which is sometimes divided into two species *C*. *mitis* and *C*. *albogularis*. The number of subspecies of *C*. *albogularis* is also under debate and is based only on differences in pelage colouration and thus far no genetic research has been undertaken on South African samango monkey populations. In this study we aim to further clarify the number of samango monkey subspecies, as well as their respective distributions in South Africa by combining molecular, morphometric and pelage data. Overall, our study provides the most comprehensive view to date into the taxonomic description of samango monkeys in South Africa. Our data supports the identification of three distinct genetic entities namely; *C*. *a*. *labiatus*, *C*. *a*. *erythrarchus and C*. *a*. *schwarzi* and argues for separate conservation management of the distinct genetic entities defined by this study.

## Introduction

The geographical distribution of the arboreal guenon *Cercopithecus albogularis* ranges from central and eastern to southern Africa where it occurs in different evergreen forest types including rainforest, Afromontane and riparian forests, as well as swamp and coastal forests [1]. The species belongs to the highly polytypic *Cercopithecus nictitans* group [2] which is sometimes divided into two species *C*. *mitis* and *C*. *albogularis*. Groves [3,4] recognises both species and uses the classification of *C*. *albogularis* for individuals distributed from Ethiopia to South Africa and occurring in South and East Democratic Republic of Congo and North West Angola. Groves classifies *C*. *mitis* as those individuals found from the Congo-Oubangui River System to East African Rift Valley, as well as in Northern Angola and North Western Zambia. The splitting of the group into two separate species by Groves is based on differences in pelage colourations. Napier [5] followed by Kingdon [6] and Grubb [2] do not recognise *C*. *albogularis* as a separate species. Furthermore the International Union for Conservation of Nature (IUCN) has not yet recorded *C*. *albogularis* as a separate taxon and *C*. *mitis* is listed as least concern [7]. No genetic analysis has been done to date to support one or the other classification and in this paper we follow Groves [3,4].

**Fig 1:**
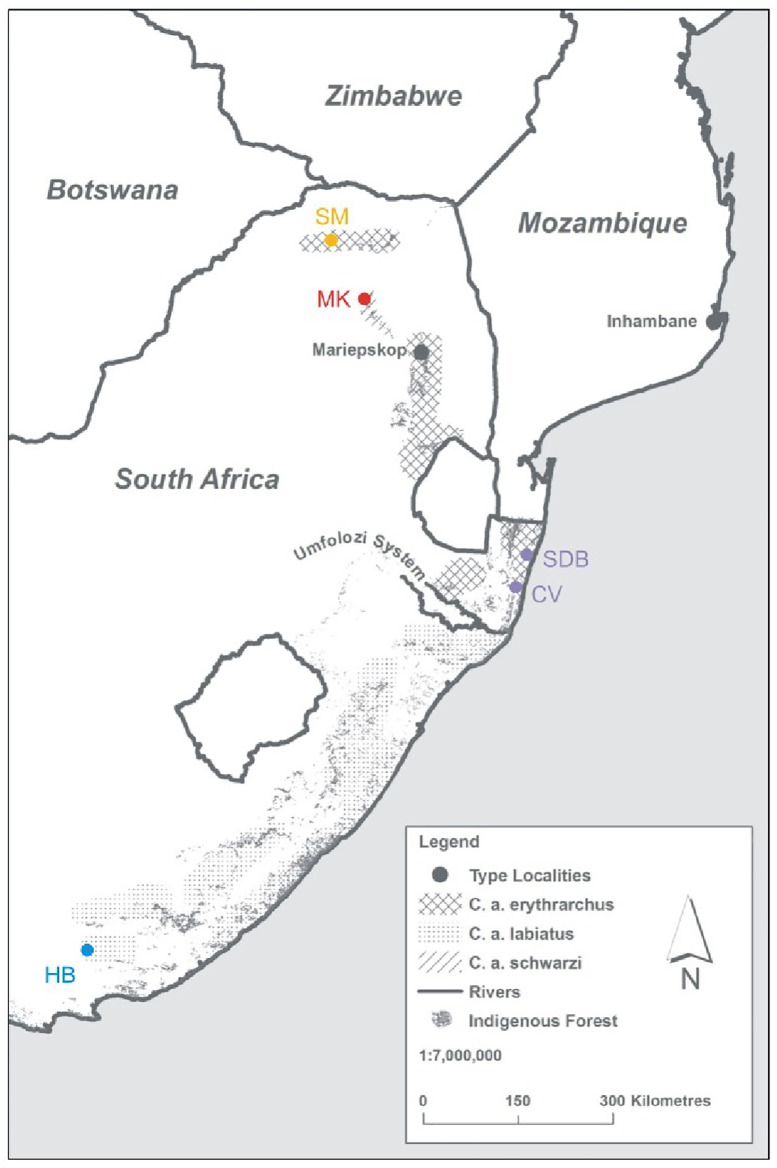
Sampling localities and distribution of proposed samango species. Geographic distribution of the proposed samango species (*Cercopithecus labiatus*, Hogsback; *Cercopithecus erythrarchus*, Cape Vidal and Sodwana Bay; *C*. *e*. *schwarzi*, Magoebaskloof and Soutpansberg Mountain) from this study. Type localities are included for *C*. *a*. *erythrarchus* (Inhambane) and *C a*. *schwarzi* (Mariepskop). No exact type locality is available for the sub-species *C*. *a*. *labiatus*. Map based on the Endangered Wildlife Trust (EWT) 2004 distribution map and modified including additional distribution data from this study. Sampling localities (SM = Soutpansberg Mountains, MK = Magoebaskloof, SDB = Sodwana Bay, CV = Cape Vidal, HB = Hogsback) are shown in relation to indigenous forests (DWAF 2004) and the Umfolozi System (Black and White Umfolozi Rivers).

The number of subspecies of *C*. *albogularis* recognised in South Africa (locally referred to as "samango monkeys" from the Zulu name iNsimango) is also disputed. Meester [8] followed by Grubb [2] recognise two subspecies namely *C*. *a*. *labiatus* [9] (type locality: South Africa), and *C*. *a*. *erythrarchus* [9] (type locality: Inhambane, Mozambique). Contrary to this, Roberts [10] followed by Dandelot [11] and Groves [3,4] recognise an additional third samango monkey subspecies in South Africa, namely *C*. *a*. *schwarzi* [10] (type locality: Mariepskop, South Africa). The present distribution of *C*. *a*. *labiatus* and *C*. *a*. *erythrarchus* is closely correlated with the distribution of Afromontane, Scarp and Indian Ocean coastal belt forests in southern Africa and the two subspecies do not overlap in their distribution [9]. *Cercopithecus a*. *labiatus* is distributed from the Pirie Forest in the Eastern Cape Province north-eastwards to the midlands of the KwaZulu-Natal Province [12]. *Cercopithecus a*. *erythrarchus* occurs from northern KwaZulu Natal through eastern Mpumalanga and central and eastern Limpopo in South Africa, through Zimbabwe and Mozambique up to Malawi (Fig. 1) [12]. The border between both subspecies appears to be at the St. Lucia and Umfolozi systems [9]. The distribution of *C*. *a*. *schwarzi* is typically from the Pilgrims Rest District, but also from Woodbush and intervening territory in the Eastern Transvaal (Mpumalanga) [3,10] (Fig. 1). Only the sub-species’ *C*. *a*. *labiatus* and *C*. *a*. *erythrarchus* are recognised by both the latest national [12] and international conservation assessments (IUCN) [7]. In South Africa the subspecies *C*. *a*. *labiatus* is listed as Endangered as it is considered endemic to South Africa whereas the subspecies *C*. *a*. *erythrarchus* is listed as Vulnerable [12]. This national divergence is based on an assumed “rescue effect from neighbouring populations (Mozambique)” for the subspecies *C*. *a*. *erythrarchus* [12]. The species itself is listed as Vulnerable due to its patchy and highly restricted distribution which in turn can be explained by the very small size of the forest biome and its highly fragmented distribution in the country [12]. Globally, the IUCN lists the subspecies *C*. *m*. *erythrarchus* as least concern whereas *C*. *m*. *labiatus* is listed as Vulnerable [7]. Given the role that taxonomy has in determining the conservation status of the species /subspecies we believe it is vital to clarify this taxonomic issue. The current subspecies classification of samango monkeys in South Africa is based only on differences in pelage colouration and thus far no genetic research has been undertaken on South African samango monkey populations. In this study we aim to further clarify the number of samango monkey subspecies, as well as their respective distributions in South Africa by combining molecular, morphometric and pelage data.

## Materials and Methods

### Ethical and permit considerations

The project was approved by the National Zoological Gardens of South Africa’s Research and Ethics Committee (Project no. P10/27). National Threatened or Protected Species (TOPS) permits (Permit no. 0 27520 and 0 27333) as well as provincial permits (Permit no. OP2407–2013-Kwazulu Natal and OP 0 8052 A-Eastern Cape) were obtained for the capture and anaesthesia of the animals as well as the sample collection, transport and storage. Animals were not removed from the wild. They were captured and released at the same location.

### Sample collection

A total of 72 samango monkey blood and tissue samples were collected from five populations in Southern Africa (Table 1). All individuals included in the morphological analyses were also included in the microsatellite analyses for all five populations, with the exception of Hogsback, where data was unavailable for a subset of captured individuals. Samples were collected from the southernmost *C*. *a*. *labiatus* population in Hogsback (HB; -32.59526, 26.95675), and *C*. *a*. *erythrarchus* from two coastal populations in Cape Vidal (CV; -28.12329, 32.55638) and Sodwana Bay (SDB; -27.54692, 32.66939), a population along the Escarpment in Magoebaskloof (MK; -23.88859, 29.99582) and from two troops from the northernmost population in the Soutpansberg mountains (SM; -23.037881, 29.441949). See 1 for the geographic distribution of sampled populations and their acronyms. Trap-door metal cage traps (1.25m x 0.6m x 0.6m) were placed on the ground and were baited with either oranges or apples. Once secured in a trap, the samango monkeys were anesthetised with an intramuscular injection of a tiletamine/zolazepam (Zoletil, Virbac, South Africa) combination at an estimated dose of 3.5–5 mg/kg. Three milliliters of blood was collected with a syringe and needle from the caudal saphenous vein and divided into serum and EDTA blood tubes (Vacutainer,BD, United Kingdom). The blood samples were placed on ice for up to 6 hours, after which they were frozen at-20°C until analysis. Several full length hairs were plucked out from between the shoulder blades of each monkey and placed in plastic *Z*iploc bags. Small (3mm in diameter) skin and cartilage samples were obtained from the pinna of each monkey using a normal leather punch. The punch was cleaned and disinfected (F10sc disinfectant, Health and Hygiene (Pty) LTD, South Africa) between samples. The skin and cartilage samples were placed in 1.5ml eppendorf tubes containing 95% ethanol. DNA was extracted using the Qiagen DNeasy Blood and Tissue Kit (GmbH, Germany) following the extraction protocol as outlined by the manufacturer. Each animal was assigned to a gender and placed in one of three age classes, namely juvenile, sub-adult or adult based on tooth eruption where sub-adults have at least one permanent canine and adults have all four permanent third molar. Standard morphological measurements were taken (see below) before the individuals were placed in a darkened cage until they had recovered from the anaesthetic to be released back into the wild. A qualified veterinarian registered with the South African Veterinary Council (registration number D97/4000) and experienced in primate capture and anaesthesia was present at all the capture sites, administered the anaesthetic agents and ensured that animals were fully recovered prior to release.

**Table 1:** Environmental and demographic characteristics of sampled populations.

Locality	Forest type	Altitude (masl)	Annual rainfall (mm)	Wet season (min-max°C)	Dry season (min-max°C)	Number samples
Soutpansberg (SM)	Limpopo Mistbelt Forest	1267	737	October-March (13–27)	April-September (4–25)	14
Magoebaskloof (MK)	Limpopo Mistbelt Forest	1518	1112	October-March (10–22)	April-September (3–21)	5
Sodwana Bay (SDB)	KwaZulu Natal Dune Forest	16	934	October-April (17–31)	May-September (12–27)	11
Cape Vidal (CV)	KwaZulu Natal Dune Forest	24	1057	October-April (17–30)	May-September (12–25)	5
Hogsback (HB)	Amatola Mistbelt Forest	1067	898	October-March (8–26)	April-September (4–21)	37

Sample locations are arranged from northernmost (top) to southernmost (bottom). Forest type descriptions follow Von Maltitz *et al*. (2003) and climatic data for each site were acquired using the Wordclim dataset by Hijmans *et al*. (2005). masl = meters above sea level, mm = millimetres and°C = degree Celsius.

### Morphological measurements

For 101 individuals, body mass (BM, kg) was obtained using a digital hanging scale (25kg Sportsman’s digital scale, Rapala, USA), whilst the following standard measurements were obtained (in cm) using both a tape measure and calliper: neck circumference (NC), head and body length from nose-tip to base of tail (HB), tail length (TL), hind foot length, without claw (HF), ear length (EL), ear width at base (EW), canine length (CW) and nipple length (NW). Full body pictures of individuals from different sites were also taken. Measurements made using a tape measure were converted using simple linear regression equations. Apart from nipple length which was only measured in females individuals, variables were log-transformed and analysed statistically for sexual dimorphism and age variation using 2-way Analysis of Variance (ANOVA) in the largest population from Hogsback (n = 67). Owing to significant variation between the three age classes, data from adults were compared between all populations using principal component analysis (PCA). All statistical analyses were carried out using the programme PAST version 1.91 [13].

### Hair analysis

In order to assess variation in hair characteristics among the different samango populations sampled we analysed individual guard hairs of adult individuals only. As the Hogsback population was sampled at different months of the year (S1 Table) we chose five individuals per each study month to also allow for analysis of seasonal variation of hair characteristics. The number of adult individuals included for the hair analysis varied as it was depended on how many adults were captured. For each individual monkey we analysed five hairs. We analysed 34 adult individuals (20 females, 14 males) totalling 170 hairs. Hair samples were analysed with the naked eye under a 20 Watt 240 Volt halogen lamp. Hair length was measured using an ordinary ruler with millimetre divisions. Five hairs originating from the same individual were fixed on the base onto a white piece of paper using transparent tape. Analysis under a dissecting microscope proved to be unsatisfactory as the hairs did not fit in the field of view in their entire length. Furthermore, the bright LED light source of the microscope made it much more difficult to detect the subtle colour differences. Hair characteristics analysed included: colour of hair base and tip, number of bands, colour of bands, number of bands per colour, width of bands and total hair length. Bands were counted starting with the first visible light band from the base of the hair. Band length was scored using three categories: bands equal, light/dark band longer and light/dark band double the length.

**Fig 2:**
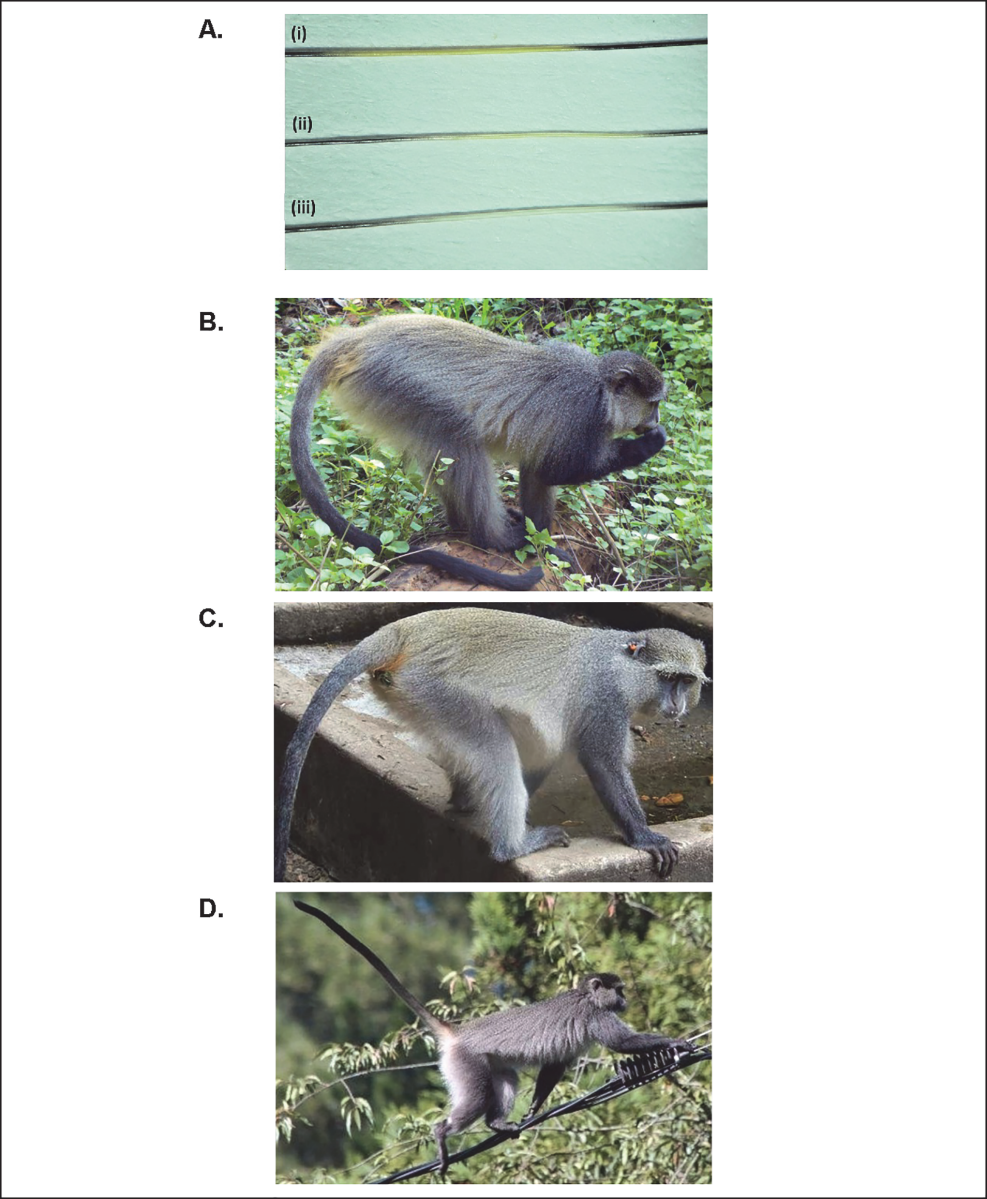
Three different colour categories identified for samango hair samples from different geographic locations. (A). (i) Cape Vidal (dark yellow category), (ii) Soutpansberg Mountain (light yellow category), (iii) Hogsback (white category).The image also illustrates the typical, alternating dark and light banding of hairs. B to D: Comparisons of pelage colouration between samango monkeys from three geographic locations. B: Adult female, Soutpansberg (Inland) (photo: Birthe Linden), C: Adult female, Cape Vidal (Coast) (photo: Joan Chalmers), D: Adult female, Hogsback (Hogsback) (photo: Kirsten Wimberger).

Hair colour was scored using three different categories: white, light yellow and dark yellow. These categories were established after comparing hairs from all different geographic locations (Fig. 2) in order to identify the range of colours (shades and extremes). Furthermore hairs of all three colour categories were fixed next to each other with transparent tape onto the white analysing sheet, constantly visible during hair analysis. In order to statistically test specifically for species/subspecies differences in hair characteristics, three geographical groups were formed: Inland (Soutpansberg Mountain and Magoebaskloof), Coast (Cape Vidal and Sodwana Bay) and Hogsback (remained on its own). All statistical analysis was carried out using the software R version 3.0.1. Full body *in situ* photographs of individuals from the three populations were compared to examine differences in pelage colouration.

### Microsatellite genotyping

All samples collected during the present study were genotyped for polymorphism at 21 microsatellite loci based on previous studies: D15S108, D14S306, D13S765, D18S536, D12S67, D9S922, D8S1106, D4S243, D5S1457, D10S611, D6S311, D5S1466, D1S518, D1S207, D3S1768, D10S1432, D7S503, D2S1326, D11S956, D11S925 and D17S1290 [14–27]. Amplification was carried out using a 15 μl reaction volume and polymerase chain reaction was conducted with PromegaGoTaqFlexi DNA polymerase, (Promega Corporation) which has a 1 x buffer containing 10 milli molar (mM) Tris-HCl (pH 9.0), 50 mM potassium chloride (KCl) and 0.1% Triton X-100. The final reaction conditions were as follows: 1 X PCR buffer, 1.5–2.5 mM MgCl_2_,200 micro molar (μM) of each 2’-deoxynucleotide triphosphate (dNTP), 10 picomol (pmol) of each of the forward and reverse primer, 1 unit (U) TaqDNA polymerase and 10–20 nano gram (ng) genomic DNA template. The conditions for PCR amplification were as follows; 5 minutes (min) at 95°C initial denaturation, 30 cycles for 30 seconds (sec) at 95°C, 30 sec at 50–65°C and 30 sec at 72°C, followed by extension at 72°C for 20 min. The PCR reaction was carried out in the BOECO TC-PRO Thermal Cycler. PCR products were pooled together and run against Genescan500 LIZ (Applied Biosystems, Inc.) internal size standard on an ABI 3130 Genetic Analyzer. Samples were genotyped using GeneMapper v. 4.0.

### mtDNA sequence analysis

The purified DNA was used as a template to amplify mitochondrial gene fragments using the Cyt B primers L14724 cgaagcttgatatgaaaaaccatcgttg and H15149 aaactgcagcccctcagaatgatatttgtcctca [28] and 16S primers 16SA cgcctgtttaacaaaaacat and 16SB ctccggtttgaactcagatca [29, 30]. Polymerase chain reaction (PCR) was carried out using a 25 μl reaction volume and was conducted with Thermo Scientifics’ DreamTaq Green PCR master mix which has a 1 x buffer containing 10 mM TrisHCl (pH 9.0), 50 mM potassium chloride (KCl) and 0.1% Triton X100. The final reaction conditions were as follows: 1 X PCR buffer, 2.5 mM MgCl_2_, 200 M of each dNTP, 5 pmol of each of the forward and reverse primer, 1 unit (U) *Taq* DNA polymerase and 20 ng genomic DNA template. PCR cycling conditions were as follows: Initial denaturation at 95°C for 5 min, annealing at 52°C for 50 sec and extension at 72°C for 1 min, 30 cycles of denaturation at 94°C for 30 sec, annealing at 50°C for 50 sec and extension at 72°C for 1 min. A final extension step of 72°C for 20 min concluded the cycling. After the initial PCR amplification, products were purified according to the Exo/Sap amplicon purification method described by Werle [31]. Cycle sequencing of the PCR products obtained was performed with the BigDye Terminator v3.1 Cycle Sequencing Kit (Applied Biosystems) according to the manufacturer’s protocol. Sequencing products were purified with the ZR DNA Sequencing Clean-Up Kit (Zymo Research) and sequenced on an ABI 3130 genetic analyser in forward and reverse. The raw sequence data were analyzed using the ABI Prism DNA Sequencer software v3.4.1.

### Analysis of nuclear genetic diversity

MICRO-CHECKER [32] was used to detect possible genotyping errors, allele dropout and non-amplified alleles (null alleles) for each microsatellite locus. Population genetic analyses were carried out at two scales: firstly, with populations defined as recognised subspecies (*C*. *a*. *labiatus* and *C*.*a*. *erythrarchus*), and secondly as four populations, largely reflecting collection localities, that were identified from spatially independent multivariate analyses of the two currently recognised subspecies. The latter populations are Hogsback (HB), Sodwana Bay and Cape Vidal combined (SDBCV), Magoebaskloof (MK) and Soutpansberg Mountains (SM). To estimate the levels of genetic diversity within populations, the mean number of alleles per locus (N_A_), observed heterozygosities (H_o_), expected heterozygosities (H_E_ and uH_E_) and deviations from Hardy-Weinberg (HW) proportions were calculated using MS TOOLKIT [33] and GENALEX version 6.5 [34,35]. Linkage disequilibrium between pairs of microsatellite loci within each population and locus was evaluated using GENEPOP 4.1.4 [36,37]. Associated probability values were corrected for multiple comparisons using Bonferroni adjustment for a significance level of 0.05. To estimate levels of divergence among populations, spatially explicit and spatially independent analyses were carried out. The former included the estimation of various fixation and differentiation indices by permutation, and *F*_ST_- and R_ST_-based analyses of molecular variance (AMOVA) [38], that were carried out in GENALEX [34,35]. Spatially independent analyses included assignment tests; principal coordinates analysis (PCoA), factorial correspondence analysis (FCA) and Bayesian cluster analysis in STRUCTURE.

### Population and regional genetic structure

The level of genetic differentiation was determined among and within the currently recognized subspecies, and the four distinctive populations. The genetic relationship between populations and individual assignments of samples was inferred via a Bayesian clustering analysis using the statistical programme STRUCTURE version 2.3.3 [39]. The programme was run without prior population information (option USEPOPINFO = 0, no LOCPRIOR), to ensure that the pre-defined populations were in agreement with the genetic data. STRUCTURE was initially run for all 72 samples with 20 replicates from K = 1–9, with a run-length of 1 million repetitions of Markov chain Monte Carlo (MCMC), following the burn-in period of 100,000 iterations. A second STRUCTURE run that included only *C*. *a*. *erythrarchus* (n = 35) samples was run with the same settings for K = 1–5. The *K* with the greatest increase in posterior probability (ΔK) [40] was identified as the optimum number of sub-populations using STRUCTURE HARVESTER [41]. CLUMPP [42] generated the average of these 20 runs for the appropriate K, and the results were visualised in DISTRUCT [43].

### Analysis of mtDNA data

Alignment and vetting of sequence data were carried out using Bioedit v7.0.9.0 [44] and FinchTV v 1.4 (Geospiza Inc.), and published sequences for closely related species were included in alignments to confirm amplification of the correct target region. Standard population genetic diversity and differentiation indices (number of haplotypes, haplotype diversity, h, nucleotide diversity, π, the average number of nucleotide differences, k and φ_ST_) were calculated using DNAsp v 5.10.01 [45] based on combined mitochondrial sequence data. Maximum Parsimony (MP), Maximum Likelihood (ML) and Bayesian MCMC phylogenetic analyses were conducted for each gene region separately, and for combined datasets. Maximum Parsimony (MP) haplotype networks [46] were generated in Network v 4.6.1.0 (www.fluxus-engineering.com) and Splitstree v 4.10 [47] (10 000 bootstrap replicates) using the default settings. Statistical model selection was carried out for each dataset in MEGA v 5.0 [48] and jModeltest [49]. ML phylogenetic trees were generated in MEGA v 5.1 [48], with branch support evaluated using bootstrap-resampling (10 000 replicates). Bayesian phylogenetic trees were estimated using MCMC in MrBayes Version 3.1.2 [50] (with one million generations, trees sampled every 100 generations) and BEAUti/BEAST v2.1.1 [51] (twenty million MCMC generations, priors as in Hart et al. (2012) and the whole tree constrained with a lognormal distribution around 6 My, mean M = 1.79, standard deviation S = 0.1), with a ten to 25% burnin. Divergence date estimates were inferred using a Bayesian approach implemented in BEAST [52]. These analyses were run separately for the two gene regions and for a combined (concatenated) dataset. The results of four independent BEAST runs were checked for adequate mixing and convergence using Tracer 1.5. Once convergence was achieved, BEAST tree files were combined using LogCombiner, summarized using TreeAnnotator 1.5.3 and visualized in FigTree 1.3.1. CorelDraw was used to edit the haplotype networks and phylogenetic trees.

## Results

### Morphometric analyses

**Fig 3:**
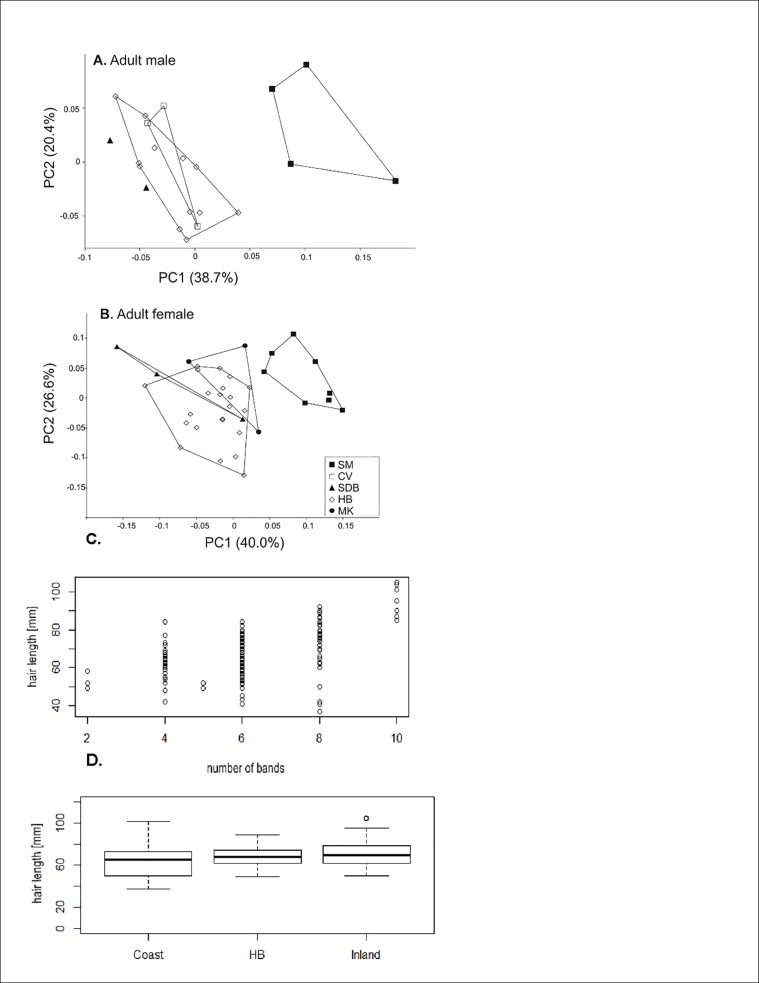
PCA of log-transformed morphometric variables. (A) adult male and (B) female samangos from five populations (closed squares = Soutpansberg Mountain (SM); open squares = Cape Vidal (CV); closed triangle = Sodwana Bay (SDB); open diamonds = Hogsback (HB); closed circles—Magoebaskloof (MK). (C) Pearson’s Correlation between hair length and number of bands per hair from all study sites. (D) Boxplots illustrating variation of hair length between three different geographic locations (Coast = Cape Vidal, Sodwana Bay, HB = Hogsback and Inland = Soutpansberg Mountain, Magoebaskloof.

As expected, age explained a significant proportion of morphometric variance (8–54%SS) in all variables except for ear length (Table 2). Significant sexual dimorphism was present in all variables except for ear length and width, neck circumference and head and body length (Table 2). Significant interaction (age-sex) terms for all variables except ear length (Table 2) signalled divergent growth projectories in males and females associated with secondary sexual dimorphism and progressively greater increases in most male morphometric variables following puberty. This is exemplified by plots of mean body mass for both sexes in the three age classes; sexual dimorphism is negligible in juvenile and subadults but very marked in adults (S1 Fig.). Based on these significant differences between age and sex classes, for subsequent analyses of population variation we excluded juveniles and subadults and analysed males and females separately. From PCA of seven log-transformed morphometric variables, both males and females showed Soutpansberg animals to be distinctly larger-sized (higher PC1 scores) than other populations, with Magoebaskloof (in females) being somewhat intermediate (Fig. 3A and 3B; S2 Table). Soutpansberg animals tended to have disproportionately longer tails than in other populations, as indicated by positive scores for PC2 on the scatterplots and positive loadings for tail length on PC2 compared with negative loadings for other variables (Fig. 3A and B).

**Table 2:** Results of 2-way ANOVA of sexual dimorphism and age variation in 67 samangos from Hogsback.

Variable	Age		Sex		Interaction	
	% SS	F(2,66)	% SS	F(2,66)	% SS	F(2,66)
**Weight (kg)**	53.8	143.2 ***	2.1	11.4**	23.8	63.5***
**Neck circumference**	42.4	41.3 ***	1.9	3.7 NS	17.0	16.6***
**Head-body**	58.7	85.8 ***	0.0002 NS	0.0008 NS	20.3	29.7 ***
**Tail**	24.1	21.5 ***	8.6	15.4 **	21.1	18.8 ***
**HF**	7.9	17.5 ***	33.8	37.5 ***	18.0	19.9 ***
**Ear length**	1.1	0.8 NS	4.6	1.6 NS	8.3	77.5 NS
**Ear width**	12.9	11.1 **	2.5	1.1 NS	7.6	3.2 *
**Canine length**	8.1	26.9 ***	34.5	57.4 ***	25.4	42.3 ***

Significance of F-values indicated as non-significant (NS)

P< 0.01 (*)

P < 0.001 (**)

P << 0.0001 (***).

SS = Sum of Squares. Value in parentheses following F-values represents degrees of freedom). Except for body mass (kg), all measurements were expressed in centimetres.

### Hair analysis

When analysing variability in hair colour, significant differences between the Soutpansberg Mountains and Magoebaskloof (Inland), coastal populations (Sodwana Bay and Cape Vidal) and Hogsback populations were found (χ² = 232.52, df = 4, p-value < 0.0001), with Inland showing light yellow bands, Coast showing mostly dark yellow bands and Hogsback showing white bands (Table 3). The relative length of black and white hair bands also differed significantly in frequency of three different categories between the three populations (χ² = 208.52, df = 6, p-value < 0.0001). Black bands were just longer than the light bands in 55 out of 56 Inland individuals, double the length of light bands in 72 out of 75 Hogsback individuals and somewhat intermediate in Coast individuals with roughly equal case where black bands were longer (21) or equal (18) (Table 3). A Pearson’s Correlation analysis of hair characteristics revealed a significant correlation between hair length and number of bands (t = 8.2603, df = 168, p-value = < 0.0001) and we found that, except in two hairs, the total number of bands was always an even number (2, 4, 6, 8, 10) and that the number of black bands and light bands was always equal in numbers (Fig. 3C). The base and tip of the hairs were found, without exception, to have the same colour in all individuals across study sites, with the base being white and the tip being black. An analysis of variance (ANOVA) of hair length showed significant differences between the three study sites (df = 2, f = 4.52, p-value = 0.0122). A post hoc Tukey HDS test showed that the Inland and Coast populations differed significantly in hair length (p-value = < 0.01); hair lengths between Hogsback and Coast as well as Hogsback and Inland did not differ significantly (Fig. 3D). Using the Hogsback population only for analysis of seasonal variation in hair length (ANOVA), we did not find significant differences of hair length between the different sampling months (df = 2, f = 0.41, p-value = 0.665). Furthermore, a t-test showed no significant differences between Hogsback males and females regarding hair length (t = -0.56, df = 70.36, p-value = 0.5801). When comparing full body in situ photographs of individuals from the three populations, pelage colouration differences are clearly visible (Fig. 2B to 2D). It can be seen that the Coast individual has an overall lighter appearance when compared to the Hogsback and Inland individuals. A marked difference regarding the darkness of individuals are the black arms of the Hogsback and Inland monkeys compared to the grey arms of the Coast monkey. The yellow wash or shine on the back is most visible and most extensive in the Coast individual and near to absent in the Hogsback individual. The ischial regions also show clear colouration differences being most prominent and orange in the Coast individual, yellow in the Inland individual and white in the Hogsback individual. Further colour differences worth mentioning are the very conspicuous white ear tufts and white underside of the tail (about the first quarter) in the Hogsback monkey compared to less obvious white ear tufts and dark tail undersides in the Inland and Coast monkeys.

**Table 3:** Total number of hairs found per colour category across the three geographic locations: Inland (Soutpansberg Mountain, Magoebaskloof), Coast (Cape Vidal, Sodwana) and HB (Hogsback).

	dark yellow	light yellow	white	black double	black longer	black equal
**inland**	1	55	/	/	55	1
**coast**	30	9	/	/	21	18
**HB**	/	11	64	72	3	/

### Genetic analysis: Populations defined as currently recognized subspecies

#### Analysis of nuclear and mitochondrial genetic diversity

In the *C*. *a*. *labiatus* (Hogsback, HB) samples, only two loci significantly deviated from HWE (loci D12S67 and D2S1326), and two loci were monomorphic (loci D11S956 and D6S311). Among *C*. *a*. *erythrarchus* individuals, the pattern was strikingly different, with 11 loci deviating from HWE. Each pair of loci in each population was tested for linkage disequilibrium (420 tests), and 81 of these were significant. However, only eight were significant within *C*. *a*. *labiatus*, and only three locus pairs showed significant evidence of linkage in both populations (loci D5S1466 and D10S1432, D10S1432 and D2S1326, D5S1457 and D2S1326). Genetic diversity was lower among *C*. *a*. *labiatus* than among *C*. *a*. *erythrarchus* in terms of, among others, the number of alleles, heterozygosity and mtDNA haplotype diversity (Table 4). The mean number of alleles per locus over the two populations ranged between 1.5 and 8, with an overall mean of 4.17 over loci and populations. All 37 *C*. *m*. *labiatus* (HB) samples exhibited at least one of 15 private alleles found at 11 loci in that population. Additionally, at least one of the 50 private alleles found among *C*. *m*. *erythrarchus* samples, i.e. alleles that were not found among *C*. *m labiatus* (HB) samples, was exhibited by all individuals (n = 35) from this population. This pattern indicates strong divergence and restricted gene flow between the currently recognized subspecies.

**Table 4:** Genetic variation estimates.

	Microsatellites						mtDNA				
Subspecies	N	N_a_	N_eA_	H_O_	H_E_	uH_E_	N	h	Hd	*π*	k
***C*. *a*. *labiatus***	**36.9**	**3.33±0.35**	**1.87±0.15**	**0.42±0.06**	**0.38±0.05**	**0.39±0.05**	**7**	**2**	**0.57**	**0.0019**	**1.71**
Hogsback (*C*. *a*. *labiatus*)	36.9	3.33±0.35	1.87±0.15	0.42±0.06	0.38±0.05	0.39±0.05	7	2	0.57	**0.0019**	**1.71**
***C*. *a*. *erythrarchus***	**31.81**	**5.00±0.41**	**2.990±0.257**	**0.48±0.04**	**0.61±0.04**	**0.617±0.039**	**21**	**6**	**0.81**	**0.004**	**3.56**
Soutpansberg (*C*. *a*. *erythrarchus*)	13.4	2.71±0.31	1.92±0.16	0.41±0.06	04±0.05	0.41±0.05	4	2	0.5	0.0017	1.5
Magoebaskloof (MK; *C*. *a*. *erythrarchus*)	4.1	2.86±0.22	2.27±0.18	0.50±0.08	0.49±0.05	0.56±0.06	5	2	0.4	0.0004	0.4
Sodwana Bay and Cape Vidal (*C*. *a*. *erythrarchus*)	14.3	3.81±0.31	2.71±0.25	0.53±0.06	0.57±0.04	0.59±0.04	12	2	0.53	0.0012	1.06
**Total (mean±SE)**	**17.2**	**3.18±0.16**	**2.19±0.1**	**0.46±0.03**	**0.46±0.03**	**0.49±0.03**	**28**	**8**	**0.87**	**0.005**	**4.37**

The sample size (N, mean across microsatellite loci and for combined mtDNA genes), number of alleles (N_a_±SE), number of effective alleles (N_eA_), observed (H_O_) and expected (H_E_) heterozygosities, and unbiased expected heterozygosity (uH_E_) over all loci; the number of haplotypes (h), haplotype diversity (Hd), nucleotide diversity (π), and average number of nucleotide differences (k) are given for the combined 16S and Cyt b dataset (911 bp). Populations are defined as the currently recognised subspecies (in bold) as well as among four populations of samango monkeys.

#### Spatially explicit analyses

Various fixation indices (F_ST_, G_ST_), corrected (standardized) fixation indices (G”_ST_) and a pure estimate of differentiation (D_EST_) were calculated to estimate genetic divergence between the two putative subspecies. Pairwise estimates of population differentiation were all high and highly significant (Table 5, P = 0.0001). The maximum possible value for G_ST_, given the dataset was G_STmax_ = 0.33±0.04. Locus D6S311 consistently showed the strongest signal of differentiation, and locus s was the only locus that did not show significant (P<0.05) differentiation among the two subspecies (locus s P = 0.479) based on G_ST_. Corrected F_ST_ (F’_ST_) and R_ST_ were estimated during AMOVA [53]. The F_ST_-based AMOVA showed that 25% of the variance in allele frequencies was explained among populations, and 14% among individuals. F_STmax_ was 0.467 and F_ST_ was estimated at 0.25 (P = 0.0001), giving an F’ST of 0.526. F_IS_ (0.188) and F_IT_ (0.391) were also high and highly significant (P<0.001), indicating strong population structure. The R_ST_-based AMOVA generated a highly significant (P<0.001) R_ST_-value of 0.157, with 16% of variance distributed among the two subspecies. Estimates based on the combined mtDNA sequence data for the two subspecies also indicated significant divergence (Table 5). Interestingly, these estimates all increased when populations were defined at a finer scale i.e. collection localities.

**Table 5:** Microsatellite-based fixation indices.

	Microsatellites						mtDNA	
	F_ST_	G_ST_	G'_STNEI_	G'_STHED_	G''_ST_	D_EST_	φ_ST_	G_ST_
**Between subspecies**	0.159±0.029	0.151±0.029	0.262±0.044	0.454±0.085	0.526±0.085	0.358±0.082	0.55	0.14
**Between populations**	0.251±0.028	0.212±0.029	0.264±0.034	0.484±0.066	0.518±0.065	0.345±0.065	0.79	0.404

Standardized fixation indices and Jost’s (D_EST_) estimate of differentiation based on the two recognized subspecies and between populations defined as the four clusters identified in the spatially independent analyses (all P<0.001). G_ST_ (Nei 1973) and φ_ST_ (Hudson et al. 1992) based on the two subspecies and four populations (both P<0.001).

#### Spatially independent analyses

**Fig 4:**
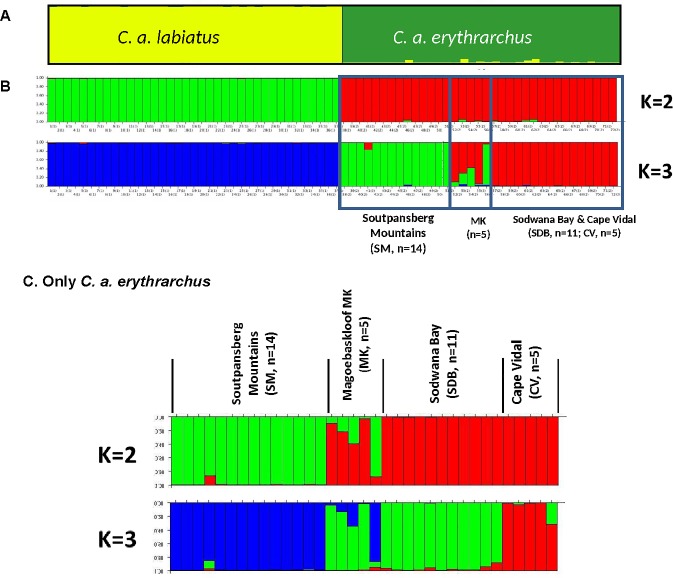
Bayesian cluster analysis in STRUCTURE. (A) The average cluster membership (over 20 runs) for K = 2 of microsatellite genotypes of *C*. *a*. *labiatus* and *C*. *a*. *erythrarchus*. (B) Example bar plots of runs where k = 2 and k = 3. (C) Example histograms from STRUCTURE analysis for K = 2 and K = 3 where only *C*. *a*. *erythrarchus* individuals are included. Each individual is represented by a single horizontal line, with lengths proportional to the estimated membership in each cluster.

Bayesian cluster analysis in STRUCTURE clearly identified the two subspecies, with members of each subspecies being highly likely to belong to that subspecies based on their multi-locus genotypes (Fig. 4A and 4B). Posterior probabilities (*Ln*) using Bayesian admixture analysis were calculated for K = 1–9, with K = 2 being identified as the most likely true K value based on STRUCTURE HARVESTER results (S2 Fig.; *K* = 2 displayed the greatest posterior probability). Results indicated two distinct clusters for *C*. *a*. *labiatus* and *C*. *a*. *erythrarchus* (Fig. 4A and 4B). All 72 individuals were correctly assigned to their subspecies based on their multi-locus genotype (S3 Fig.), indicating that the two taxa are highly distinctive.

#### Multivariate Principal Coordinates and Correspondence analyses

*C*. *a*. *labiatus* individuals from Hogsback formed a tight, distinctive cluster in the principal coordinate’s analysis and are clearly divergent from all *C*. *a*. *erythrarchus* individuals (S4 Fig.), and the forest and village troops appear to be genetically homogenous based on their multi-locus genotypes. Samples of the latter subspecies, however, appear to show higher levels of structure, and fall into three groups that largely reflect their sampling locality (S4 Fig.). Factorial correspondence analysis conducted in GENETIX showed a very similar pattern to the principle coordinates analysis in that *C*.*a*. *labiatus* individuals from Hogsback formed a tight, distinct group, and *C*.*a*. *erythrarchus* from other sampling localities were also distinct from each other, although they were clearly more similar to each other than to HB samples (S5 Fig.). However, one Soutpansberg Mountain sample (SMBT6) appears to be more closely related to individuals from Magoebaskloof (S4 Fig.).

#### Phylogenetic trees

Two distance measures based on microsatellite data were used to construct UPGMA phylogenetic trees of individuals with bootstrapping over loci (1000 replicates): DSW and Cavalli-Sforza and Edwards Dc (chord distance, S6 Fig.). Both methods produced similar results that reflected the multivariate analyses above. All *C*. *a*. *labiatus* individuals from Hogsback form their own distinctive clade (Clade A, S6 Fig.). The Soutpansberg Mountain and Magoebaskloof *C*. *a*. *erythrarchus* individuals appear to be very closely related (Clades C and D respectively, S6 Fig.), but are quite distinct from the coastal Sodwana Bay and Cape Vidal individuals (Clade B, S6 Fig.).

### Fine-scale analyses: Populations defined as collection localities

Results based on the full microsatellite dataset of 72 individuals suggest that further structure exists among the *C*. *a*. *erythrarchus* individuals sampled. A second STRUCTURE analysis was therefore run that omitted *C*. *a*. *labiatus*, which is clearly very distinct from all *C*. *a*. *erythrarchus* populations. Settings for this STRUCTURE analysis were 100 000 burnin, 1 million MCMC, admixture model, no LOCPRIOR, and 20 replicates for K = 1 to K = 5. STRUCTUREHARVESTER identified the most likely number of clusters as K = 2 (Fig. 4C). Structure results for the 20 runs (1 million) were averaged using CLUMPP, and visualized using DISTRUCT.

The two *C*. *a*. *erythrarchus* clusters strongly reflect their collection localities in the Soutpansberg Mountains, and in the coastal Sodwana Bay and Cape Vidal populations (Fig. 4C). However, those samples collected in Magoebaskloof are not as distinctive. The observed population structure appears to reflect coastal and inland forms of *C*. *a*. *erythrarchus*, with restricted gene flow between them. However, samples from Magoebaskloof (MK) resemble hybrids in that most are equally likely to have originated in either of the aforementioned coastal and inland clusters.

#### HWE, LD, genetic diversity, private alleles

Population genetic analyses were repeated with populations defined as the three *C*. *a*. *erythrarchus* clusters identified by STRUCTURE, and the Hogsback *C*.*a*. *labiatus* population. Where deviations from HWE remained the same for *C*. *a*. *labiatus*, three loci were monomorphic in the Soutpansberg Mountain (SM, loci D6S311, D10S1432, D11S956) population, and two deviated from HWE (D4S243 and D1S207). Loci D10S1432, D1S207 and D5S1466 deviated from HWE in the MK population, and D11S956 was monomorphic. Loci D2S1326, D10S1432, D5S1466, D10S611, D8S1106 and D12S67 deviated from HWE in the SDCV population. 37 of the 840 pairwise linkage tests were significant, but no locus pair showed significant linkage in all four populations. Over all four populations, GENEPOP identified six pairs of loci as significantly linked (S3 Table). Although 171 pairwise comparisons were affected by missing data and monomorphic loci, it does not appear that linkage disequilibrium will influence these results. Across all four populations and all loci, the mean number alleles (Na) was 3.4 and mean observed heterozygosity (Ho) was 0.46 (Table 4). Observed heterozygosity and number of alleles was highest in the SDB and CV population. The highest haplotype and nucleotide diversity was found among *C*. *a*. *labiatus* individuals from Hogsback (Table 4) and the lowest among *C*. *a*. *erythrarchus* from Magoebaskloof (Table 4). All *C*. *a*. *labiatus* individuals still exhibited at least one of the 12 private alleles in that population, but the each of the *C*. *a*. *erythrarchus* clusters also exhibited private alleles: SM exhibited one private allele (exhibited by one individual, SMN8), MK had five private alleles (at least one of which is exhibited by all samples). The combined SDB and CV populations exhibited 20 private alleles, and all individuals except one (SDB 3) exhibited at least one of these private alleles.

#### Spatially explicit analyses

Most indices of overall population structure increased compared to the previous analysis comparing only the two subspecies (Table 5).

#### AMOVA-based estimates of population differentiation

Overall, F_ST_ based on AMOVA was 0.29 (P<0.001), F_STmax_ was 0.50, and corrected F_ST_, therefore, equals 0.58. Also, 29% of variance is explained among the four populations, and 9% among individuals. Pairwise F_ST_ values estimated during AMOVA (Table 6) show that the HB population is consistently significantly and highly differentiated from all other populations, and that within *C*. *a*. *erythrarchus*, Soutpansberg Mountain population is most different from the Sodwana Bay and Cape Vidal population. The analogous analysis of the two subspecies produced an F_ST_ of 0.24 (P = 0.0001), which is lower than the estimate between these two *C*. *a*. *erythrarchus* populations. Importantly, all populations are significantly different from one another. Overall R_ST_ was 0.23 (P<0.001), and pairwise R_ST_-values also indicate high, and highly significant, differentiation among the four predefined populations. The largest differences are again between *C*. *a*. *labiatus* and each of the three *C*. *a*. *erythrarchus* subpopulations (all pairwise R_ST_>0.15, Table 6), although the latter are also significantly different from each other. Analogous results based on the combined mtDNA dataset corroborated the finding of significant population differentiation (Table 7, all P<0.001), although the pattern of divergence was different.

**Table 6:** AMOVA-based estimates of population differentiation based on microsatellites.

Population	HB	SM	MK	SDBCV
Hogsback (HB, *C*. *a*. *labiatus*)	-	0.000 (0.000)	0.000 (0.000)	0.000 (0.000)
Soutpansberg Mountains (SM; *C*. *a*. *erythrarchus*)	0.389 (0.156)	-	0.000 (0.001)	0.000 (0.000)
Magoebaskloof (MK; *C*. *a*. *erythrarchus*)	0.371 (0.752)	0.221 (0.320)	-	0.000 (0.000)
Sodwana Bay and Cape Vidal (SDB, CV; *C*. *a*. *erythrarchus*)	0.304 (0.375)	0.251 (0.146)	0.163 (0.265)	-

Pairwise F_ST_ (R_ST_ values indicated in brackets) values estimated during AMOVA (below diagonal) and the associated probabilities (above diagonal).

**Table 7:** AMOVA-based estimates of population differentiation based on mtDNA.

Population	HB	SM	MK	CV	SDB
Hogsback (HB, *C*. *a*. *labiatus*)	-	0.65	0.86	0.85	0.85
Soutpansberg Mountains (SM; *C*. *a*. *erythrarchus*)	0.29	-	0.85	0.66	0.82
Magoebaskloof (MK; *C*. *a*. *erythrarchus*)	0.33	0.38	-	0.97	0.97
Cape Vidal (CV; *C*. *a*. *erythrarchus*)	0.51	0.63	0.66	-	1
Sodwana Bay (SDB; *C*. *a*. *erythrarchus*)	0.54	0.64	0.69	1	-

Pairwise population ϕ_ST_ (above the diagonal) and G_ST_ (below the diagonal).

### Spatially independent analyses

#### Population assignment test

Overall, 99% of individuals were correctly assigned to their population. The only exception was one individual from Magoebaskloof (MK5), which was assigned to the Soutpansberg Mountain population.

#### Phylogenetic analysis of mtDNA data

**Fig 5:**
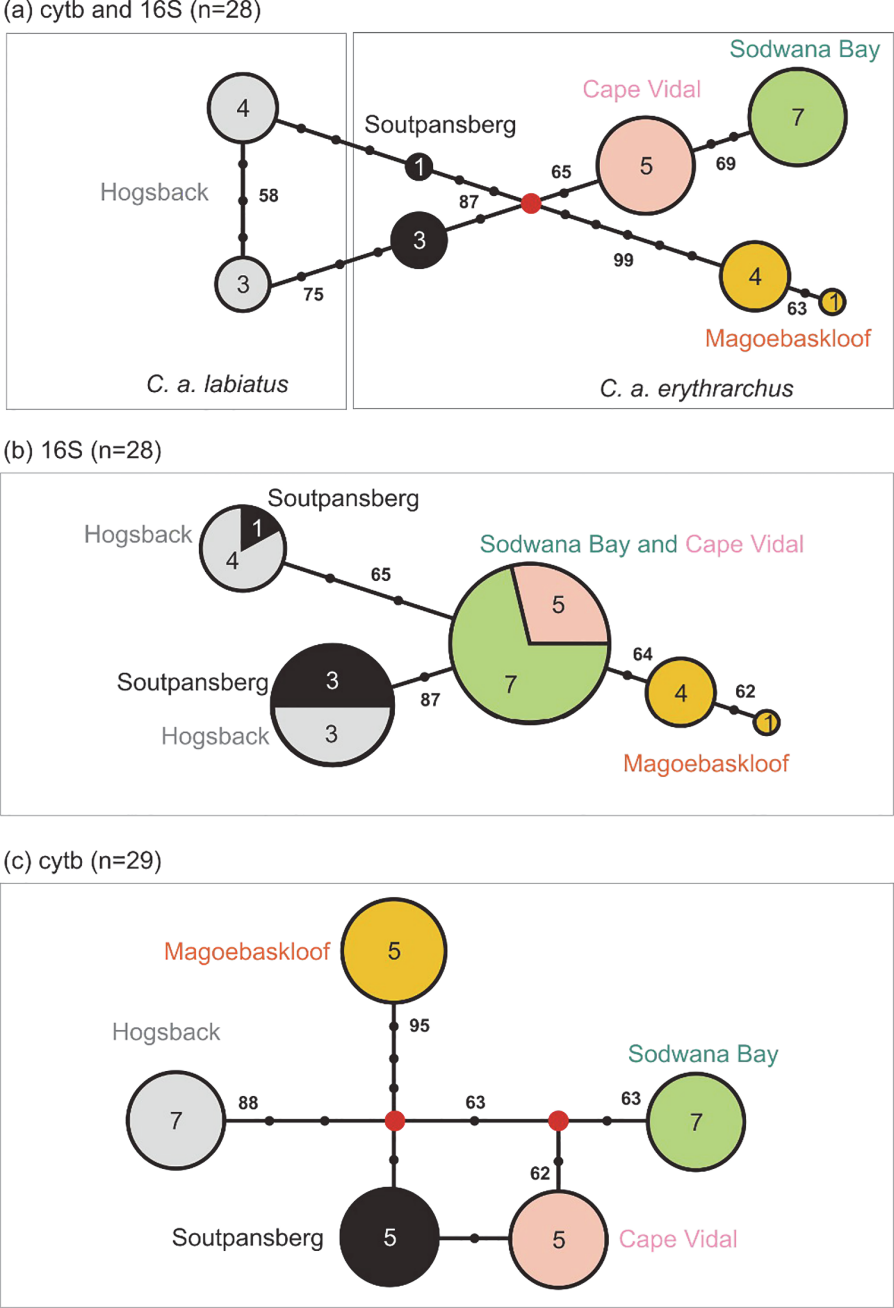
Maximum Parsimony reduced-median haplotype networks. (a) Combined 16S and cyt b data, (b) 16S data and (c) cytochrome b data. The sizes of circles, and numbers in circles, represent the number of individuals sharing a haplotype, red circles represent missing haplotypes, and tick marks along connecting lines are nucleotide changes. Numbers in bold along the connecting lines are bootstrap support.

A total of 911 bp of mtDNA sequence data (416 bp of cyt b and 495 bp of 16S; Genbank accession numbers KP120559 to KP120615) were generated in 28 samples (HB = 7, SDB = 7, CV = 5 and SM = 4 and MK = 5). Each gene region contained five haplotypes, and when analysed together, there were a total of eight haplotypes (haplotype diversity = 0.87). MP-based haplotype networks largely reflect the pattern of divergence estimates from analyses of population differentiation (Fig. 5). Haplotypes are largely restricted to single populations, and are therefore not likely to represent numts (nuclear copies of mitochondrial genes). Interestingly, some individuals from Hogsback (*C*. *a*. *labiatus*) share 16S haplotypes with individuals from the SM population (*C*. *a*. *erythrarchus*). The pattern of reticulate evolution reflected in the haplotype networks cannot be represented as a bifurcating phylogenetic tree, but is evident as reduced nodal support in the ML and Bayesian trees (S7–12 Figs.). The combined ML analysis placed *C*. *a*. *labiatus* as the basal lineage separate from the *C*. *a*. *erythrarchus*. In addition, ML analysis did not favour a monophyletic *C*. *a*. *erythrarchus* group but rather split the samples into sister groups as shown in S9 Fig. This pattern was identical to that of the cyt b Bayesian tree (S11 Fig.). However, when 16S is incorporated into analyses, the shared haplotypes between HB and SM changes the tree topology (S12 Fig.). The Bayesian tree constructed in BEAST (S13 Fig.) reflects the complexity of the relationships within *C*. *a*. *erythrarchus* and the reticulate nature of these relationships in that it places the SM population as sister to CV. It also clearly shows the distinctiveness of the HB, MK and SDB populations.

## Discussion

### Morphological variation

Morphometric variables in isolation, could not distinguish the two genetic lineages; *C*. *a*. *labiatus* and *C*. *a*. *erythrarchus* described by Meester [8] followed by Grubb [2]. Instead, the Soutpansberg (SM) and northern Escarpment (Magoebaskloof, MK) populations currently classified as *C*. *a*. *erythrarchus* are distinctly heavier and larger in most body metrics than both the Hogsback (Eastern Cape, HB) population of *C*. *a*. *labiatus* as well as the coastal forest populations of *C*. *a*. *erythrarchus* from Sodwana Bay (SDB) and Cape Vidal (CV) in KwaZulu-Natal. Body size in many animals is known to be strongly influenced by environmental factors resulting in well established “rules” of geographic variation, e.g. towards increasing body size at higher latitudes and elevations (Bergman’s Rule) [54]. Whilst Bergmann’s Rule might explain the larger body size of SM animals compared with those from coastal populations (SDB, CV), the data from HB are anomalous since both the high elevation, high latitude and similar climatic conditions to the Soutpansberg of this site would predict the largest or at least equal body size. It may be that nutritional constraints or other environmental factors limit body size in the Hogsback population. We found clear pelage colour polymorphism in South African samango monkeys and were able to identify three distinct geographic colour morphs: Hogsback, Inland (Soutpansberg, Magoebaskloof) and Coast (Cape Vidal, Sodwana Bay). This finding supports the presence of three sub-species as first proposed by Roberts [10] and as currently accepted by Groves [3]. Within the three geographic colour morphs we found that the HB individuals (currently classified as *C*. *a*. *labiatus*) show hardly any yellow colouration, neither on the back nor in the ischial region (observations also made by both Roberts [10] and Groves [3,4] for *C*. *a*. *labiatus*) and clearly setting them apart from Inland (SM, MK) and Coast (SDB, CV) individuals. The most pronounced difference between Coast individuals and all the other populations found was their generally lighter pelage colour, the prominent and comparatively extensive yellow wash on the back and the orange ischial region. These results are very similar to those of Roberts [10] and Groves [3] who found that the coastal C. a. *erythrarchus* individuals had the most conspicuous reddish colouration in the ischial region. The overall lighter appearance of the pelage in Coast individuals found in our study could be explained by the fact that in 46% of Coast hairs analysed the light bands were equal to the length of the dark bands as opposed to Hogsback and Inland individuals where black bands were either double the length (Hogsback) or longer than light bands (Inland), resulting in an overall darker appearance. The conspicuously black arms found in both Hogsback and Inland individuals further increase the effect of this dark appearance. Roberts [10] and Groves [3] found that the main colour difference between *C*. *a*. *schwarzi* and *C*. *a*. *erythrarchus* is that the ischial region is buffy yellow (Roberts) or reddish (Groves) in *schwarzi* but not as reddish as it is in coastal *C*. *a*. *erythrarchus* (Roberts). This description compares well to the pelage colouration we observed for the Inland individuals which had a yellow rather than orange (Coast) ischial region. The Inland population was made up of individuals from Magoebaskloof and the Soutpansberg as they showed the same hair characteristics. Morphological results from our study could not be compared to Meester [8] and Grubb [2] as they do not give any other reasoning rather than distribution to delineate the two subspecies, *erythrarchus* and *labiatus*.

### Nuclear genetic diversity and population structure

Across the whole dataset of 72 individuals, the number of alleles detected per microsatellite locus ranged from two to 11, with a mean of four, and a mean expected heterozygosity (H_E_) of 0.5. The total H_E_ was 0.59. When the dataset was subdivided to reflect the two currently recognised subspecies, *C*. *a*. *labiatus* and *C*. *a*. *erythrarchus*, the former showed lower levels of genetic diversity in terms of number of alleles and heterozygosity (H_E_ = 0.38) and fewer deviations from HWE relative to the latter (H_E_ = 0.62). The H_E_ value for *C*. *a*. *labiatus* falls into the lower range of what has previously been reported for Neotropical primate species [55], for example, among others, Capuchin monkeys (*Cebus apella*, H_E_ = 0.378) and Squirrel monkeys (H_E_ = 0.239). However, among *C*. *a*. *erythrarchus*, genetic diversity estimates are among the highest described for primate species, for example red howler monkeys (*Alouatta seniculus*, H_E_ = 0.638) and brown woolly monkeys (*L*. *lagotricha*, H_E_ = 0.54). The strong deviations from HWE observed among *C*. *a*. *erythrarchus* samples seem to be as a result of population subdivision i.e. the Wahlund effect, as inbreeding and strong genetic drift would have resulted in lower heterozygosity and numbers of alleles [55]. Also, spatially independent analyses detected some structure among samples belonging to *C*. *a*. *erythrarchus*. In terms of genetic structure and divergence between the currently recognised subspecies, we found strong evidence for isolation based on mutually exclusive private alleles in each subspecies i.e. all the individuals belonging to each taxon exhibited alleles that were not found in the other taxon. This pattern was reflected in the highly significant pairwise estimates of population differentiation (e.g. G_ST_ = 0.146, P<0.001; φ_ST_ = 0.55), which were as high, or higher, than those reported for currently recognised primate species [55]: for example, among *Cebus* species (G_ST_ = 0.08 to 0.11), *Ateles* species (G_ST_ = 0.105 to 0.123) and *Aloutta* species (G_ST_ = 0.189 to 0.259). The R_ST_-value estimated during AMOVA, however, was lower between samango subspecies (R_ST_ = 0.15, P<0.001) than those reported for other primate species e.g. *Cebus* species pairwise R_ST_-values ranged from 0.26 to 0.33.

Spatially independent analyses of the nDNA dataset consistently identified strong subdivision between the two currently recognized subspecies, and also within the *C*. *a*. *erythrarchus* samples. The finer scale structure within this taxon co-reflect the collection locality of individuals in the Soutpansberg Mountains and Magoebaskloof (inland), and coastal populations in Sodwana Bay and Cape Vidal. Bayesian clustering analysis indicates that the coastal and inland forms are distinctive based on their multi-locus genotypes, but that the Magoebaskloof population that falls geographically between the coastal and Soutpansberg populations, resembles both inland and coastal forms, indicating a possible “hybrid zone” between these two population units.

Expected heterozygosity within these *C*. *a*. *erythrarchus* sub-populations ranged from 0.34 in the Soutpansberg Mountains to 0.58 in the coastal population (Sodwana Bay and Cape Vidal). Importantly, population differentiation estimates based on the four predefined populations increased compared to analyses comparing only the two currently recognised subspecies. This, together with the results of the finer scale spatially independent analyses e.g. the assignment test (99% correct assignment) and Bayesian clustering, indicate strong structure among coastal and inland forms of *C*. *a*. *erythrarchus*, and exceptionally strong differentiation between these populations and that of *C*. *a*. *labiatus*.

### Mitochondrial population structure

Mitochondrial DNA analysis generated discordant patterns from nuclear DNA in terms of reconstructed relationships among groups. In contrast to both phenotypic descriptions and nuclear DNA, the HB and SM individuals were found to be closely related based on 16S and the SM and coastal populations (SDB and CV) were more similar based on cyt b. The coastal and MK populations were differentiated into two separate groups, and MK was consistently distinctive from all other populations. Discordance between mtDNA and patterns observed for nuclear genomes has been widely reported and has been found to be the highest in mammals [56]. Discordance may be due to inadequate data, homoplasy, nucleotide composition or hybridisation [57]. Discordant gene trees have been previously reported in Colobine monkeys where the authors attributed their findings to ancestral hybridisation [57]. In this study, discordance is most likely due to geographic isolation of HB (*C*. *a*. *labiatus*) and SM (*C*. *a*. *erythrarchus*) populations followed by secondary contact which could occur either via hybridisation, due to human mediated interventions or due to sex-biased dispersal. There is evidence that females are philopatric while males leave their troops before sexual maturity at 6–8 years old [58–60]. However, further sampling would have to be conducted in the intervening areas to properly ascertain whether clinal variation occurs between these two populations. In this case, a hybrid zone between these populations may be evident.

### Phylogeography

Paleoclimatic data of the late Quaternary (last 150.000 years) was used by Lawes [9] to explain the current distribution of samango monkeys and forests in South Africa. *Cercopithecus a*. *labiatus* is suggested to represent the first dispersal event from East Africa along Afromontane forests down into South Africa before the last glacial maximum (LGM) whereas *C*. *a*. *erythrarchus* is thought to have entered South Africa in a second dispersal event, after the LGM within the last 12.500 years, primarily along the expanding coastal belt forest on the Mozambique seaboard. *Cercopithecus a*. *labiatus* was until then in all likelihood the only representative of the species present in southern Africa prior to and during the last glacial maximum [9]. Our study shows that *labiatus* and *erythrarchus* diverged about 1.7 Mya (1.6–2.6 Mya) during the mid-Pleistocene. During this period the subtropical African climate periodically oscillated between markedly wetter and drier conditions, with step-like increases of variability and aridity near 2.8 Ma, 1.7 Ma, and 1.0 Ma and also suggesting more varied and open habitats after 1.8 Ma [61]. These wet-dry oscillations will have had a marked influence on the extent and continuity of forest habitat on the continent and DeMenocal [62] suggests that they offered discrete opportunities for ecologic fragmentation and genetic isolation. The subspecies *labiatus* was likely isolated by retreating forests during one of the drier Pleistocene periods, indicating that some forest refugia remained south of the Umfolozi system, not only throughout the LGM but also throughout the mid Pleistocene. There is also no genetic indication of secondary hybridisation with later radiations of the species which suggests that a geographic barrier of some sort, such as the Umfolozi system, as suggested by Lawes [9], will have prevented subsequent radiations dispersing further south. Our data supports the theory of separate radiation events of the species into southern Africa. However, the suggestion of only two recent radiation events suggested by Lawes [9] is possibly, considering our results, a bit too simplistic. The first radiation of what we currently refer to as *labiatus* must have, according to our results, occurred prior to the mid-Pleistocene and it thus seems more likely that several older Pleistocene radiation events occurred before the LGM. There is also no reason to assume that gallery forests of river systems (e.g. Limpopo system) connecting forests did not play a role in the radiation of the species. Our results indicate that there are genetic as well as morphological differences between the coastal and inland *erythrarchus* populations in South Africa. Future sampling of inland and coastal populations from Zimbabwe (inland) and Mozambique (coast and inland) further north will show if this might be a general pattern within the species range and also give additional insights about possible radiation routes and the number of radiation events.

### Taxonomic conclusions

Our study gives the most comprehensive insights to date into the taxonomic description of samango monkeys in South Africa. Spatially independent analyses of the nDNA dataset and data based on pelage colour consistently identified strong subdivision between the two currently recognized subspecies, and also within the *C*. *a*. *erythrarchus* samples. The reticulate evolutionary pathway evident in the mtDNA haplotype networks, discordant from nDNA, likely represents an ancient pattern of hybridisation or introgression among samango monkey populations, and highlights the complex evolutionary history of this group. Differences in morphology and nDNA markers, however, suggest that these populations are on distinct evolutionary trajectories. Our data thus support the two recognised subspecies namely; *C*. *a*. *labiatus* and *C*. *a*. *erythrarchus*. Within *C*. *a*. *erythrarchus* two lineages should be proposed. The Soutpansberg is currently classified as *C*. *a*. *erythrarchus* by Friedmann and Daly [12] and is geographically not explicitly mentioned in any samango subspecies descriptions by either Roberts [10] or Groves [3]. However, as Magoebaskloof geographically falls under the distribution range described for *C*. *a*. *schwarzi* by Roberts and Groves and as the Soutpansberg and Magoebaskloof show the same pelage characteristics, the Soutpansberg population should more accurately be classified as *C*. *a*. *schwarzi*. Whereas, the costal populations (CV and SDB) may be assigned to *C*. *a*. *erythrarchus* (type locality Inhambane on the coast of Mozambique). However, the taxonomy of the Magoebaskloof population remains unresolved. This population is highly divergent based on mtDNA sequence data, but is relatively uniform genetically (nDNA) and phenotypically (morphometrics and pelage colour) to the SM population (*C*. *a*. *schwarzi*). However, it is unclear whether this population can be attributed to *C*. *a*. *schwarzi* or if it is a hybrid zone or a distinct lineage. Therefore, the above hypothesis should be tested by further sampling in intermediate localities and adjacent countries to properly ascertain whether clinal variation occurs throughout the range of these taxa, or if they are isolated, as it currently unclear whether these lineages are distinct species or sub-species. Additional markers (such as X- and Y-chromosomes and mobile elements) are also required to fully resolve the taxonomy of this species and test the validity of *C*. *mitis* versus *C*. *albogularis*. The final taxonomic outcome is dependent on choice of species concept. According to the Phylogenetic Species Concept (PSC), the three lineages are diagnosable (on three independent characters) and therefore represent species. The PSC has gained widespread acceptance although its application to large mammals has been recently questioned by some conservationists and defended by systematists [63–70]. Regardless of the taxonomic outcome, our data argue for separate conservation management of the three distinct genetic entities defined by this study, as Evolutionarily Significant Units (*C*. *a*. *erythrarchus* and *C*. *a*. *labiatus*) and Management Units (coastal and inland populations of *C*. *a*. *erythrarchus*) following the definitions of Moritz [71]. Distinct genetic entities need to be conserved to protect the loss of genetic diversity, as this diversity is an essential part of biodiversity conservation.

## Supporting Information

S1 Fig Growth in mean body mass in male and female samango monkeys from Hogsback. (TIFF)

S2 Fig Output from STRUCTURE HARVESTER showing (A) Probability (-LnPr) of K = 1 to 9 averaged over 20 runs. (B) Delta K values for real population structures of K = 1–9. Output from STRUCTURE HARVESTER showing (C) Probability (-LnPr) of K = 3 and (D) Delta K values for real population structures of K = 3.
(TIFF)

S3 Fig Log-likelihood plot for all 72 individuals indicating their log-likelihood of being assigned to their own subspecies, or the other (log-likelihoods on axes are negative values and the highest (least negative) value indicates the most likely population). (TIFF)

S4 Fig Principal coordinates analysis showing (a) the subspecific identity of samples and (b) their collection localities. (TIFF)

S5 Fig Spatially independent correspondence analysis incorporating all 72 samples of samango monkey. (a) and (b) are different projections of the same results. Individuals are colour-coded according to their collection locality (HB, Hogsback; SBCV, Sodwana Bay and Cape Vidal; MK, Magoebaskloof; and SM, Soutpansberg Mountains).
(TIFF)

S6 Fig Individual unrooted UPGMA phylogenetic trees based on two measures of genetic distance: (a) Cavalli-Sforza and Edwards *Dc* (chord distance) and (b) Dsw. (TIFF)

S7 Fig Maximum likelihood phylogenetic tree based on the 16S sequence data for 28 individuals. Numbers at the nodes are bootstrap support.
(TIFF)

S8 Fig Maximum likelihood tree based on cytochrome b sequence data for 29 individuals. Numbers at the nodes are bootstrap support.
(TIFF)

S9 Fig Maximum-likelihood phylogenetic tree based on 911 bp of mtDNA sequence data (16S and cyt b). Numbers at the nodes are bootstrap support (<50% not shown).
(TIFF)

S10 Fig Bayesian Phylogenetic tree based on 16S sequence data for 28 individuals. Numbers at the nodes are Bayesian posterior probabilities.
(TIFF)

S11 Fig The Bayesian 50 percent majority rule consensus tree based on cytochrome b sequence data (416 bp and 29 individuals). Numbers at the nodes are posterior probabilities.
(TIFF)

S12 Fig Bayesian phylogenetic tree based on the combined, concatenated (partitioned) mtDNA sequence data (16S and cyt b). Numbers at the nodes are posterior probabilities.
(TIFF)

S13 Fig Bayesian analysis in BEAST, showing the estimated dates of divergence of *Cercopithecus* subspecies (Genbank accession numbers are given for each sequence. (TIFF)

S1 Table Hairs analysed from the five sampling sites detailing the sampling month, the number of males and females, the total number of individuals and the total number of hairs analysed per site with a total of five hairs analysed per individual. (DOCX)

S2 Table Variable loadings from PCA of seven variables in 21 adult male and 36 adult female samango monkeys from four populations. (DOCX)

S3 Table The six significant pairs of loci identified by LD analysis in GENEPOP. (DOCX)
