# Correction: New Insights into Samango Monkey Speciation in South Africa

**DOI:** 10.1371/journal.pone.0129988

**Published:** 2015-06-03

**Authors:** Desiré L. Dalton, Birthe Linden, Kirsten Wimberger, Lisa Jane Nupen, Adrian S. W. Tordiffe, Peter John Taylor, M. Thabang Madisha, Antoinette Kotze

The affiliation for the fourth author is not indicated. Lisa Jane Nupen is affiliated with: 1 National Zoological Gardens of South Africa, Pretoria, South Africa, 6 Percy FitzPatrick Institute, Department of Biological Sciences, University of Cape Town, Rondebosch, South Africa, 8 Centre for Conservation Science, National Zoological Gardens of South Africa, Pretoria, Gauteng, South Africa.
