# Supplementary figures and images for: New Insights into Samango Monkey Speciation in South Africa
Source: PLoS One. 2015 Mar 23;10(3):e0117003. doi: 10.1371/journal.pone.0117003 (PMC4370472; doi:10.1371/journal.pone.0117003)

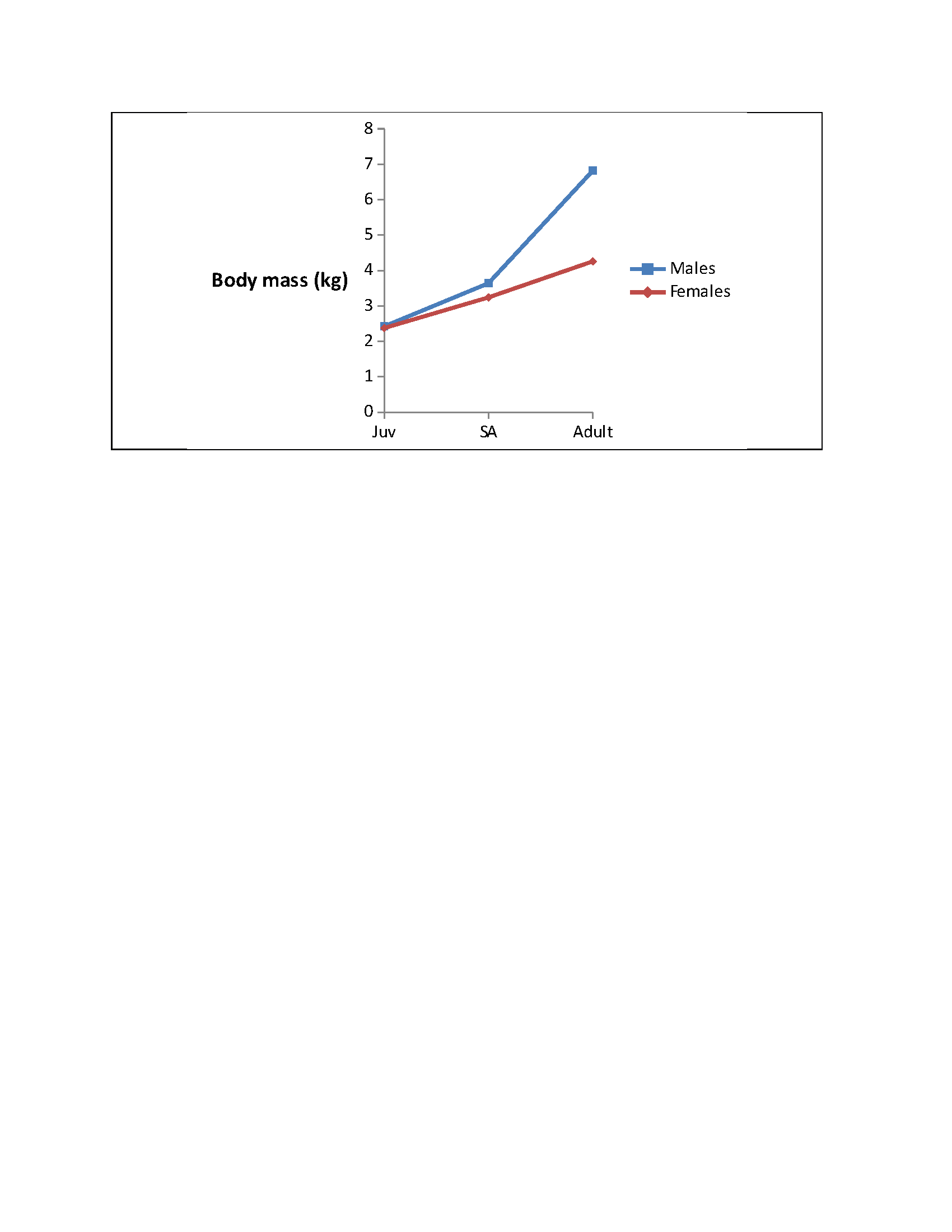

Supplement: S1 Fig — (TIFF) [file pone.0117003.s001.tiff]

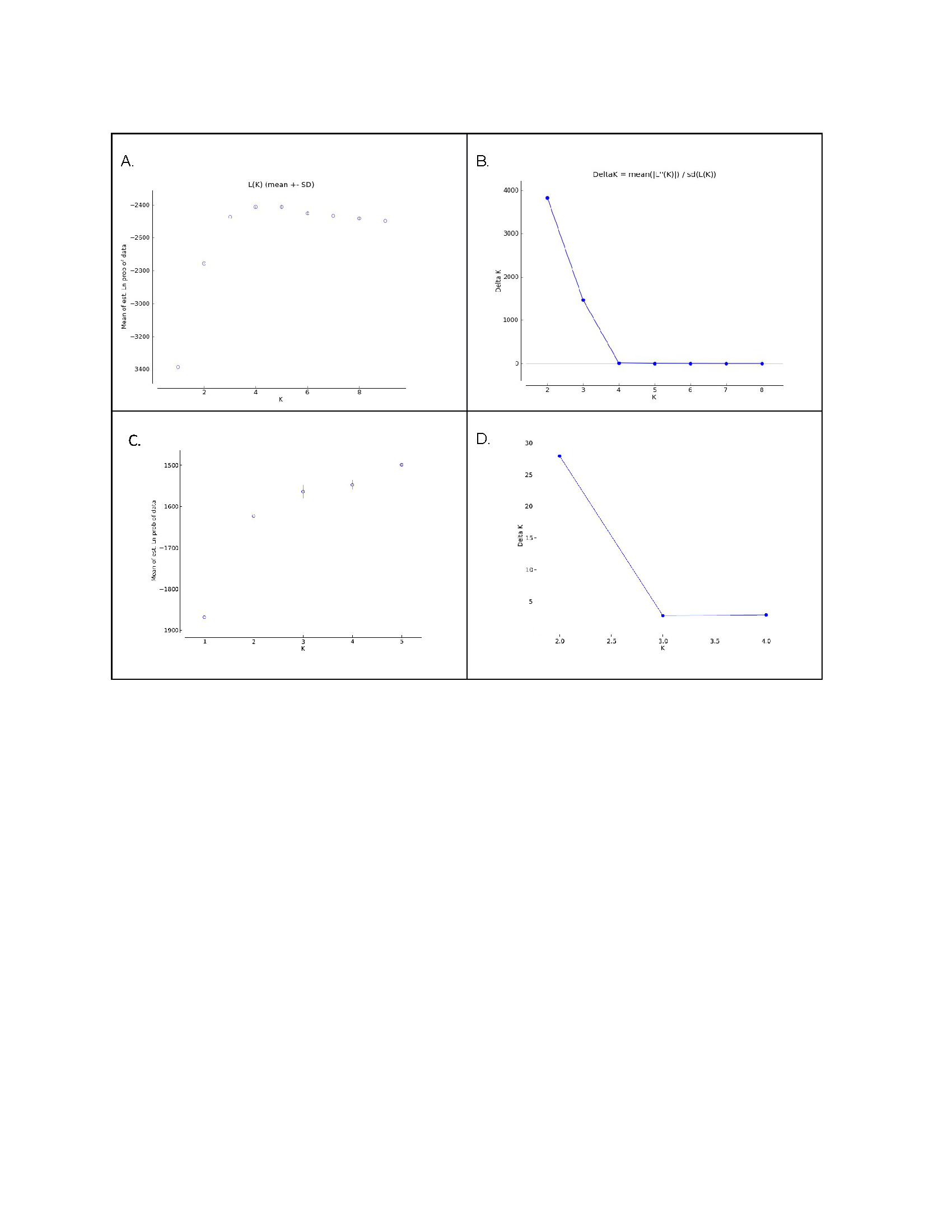

Supplement: S2 Fig — (B) Delta K values for real population structures of K = 1–9. Output from STRUCTURE HARVESTER showing (C) Probability (-LnPr) of K = 3 and (D) Delta K values for real population structures of K = 3. (TIFF) [file pone.0117003.s002.tiff]

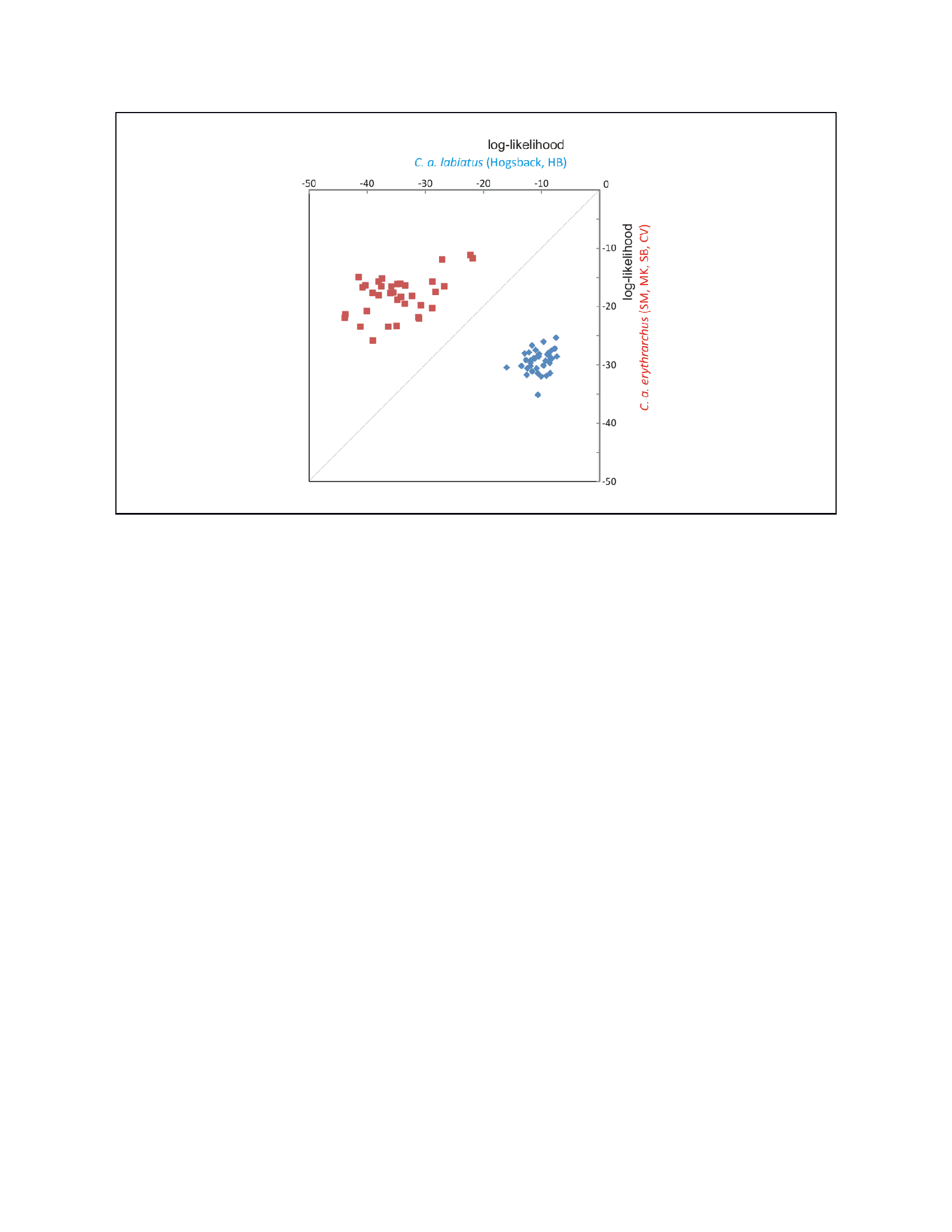

Supplement: S3 Fig — (TIFF) [file pone.0117003.s003.tiff]

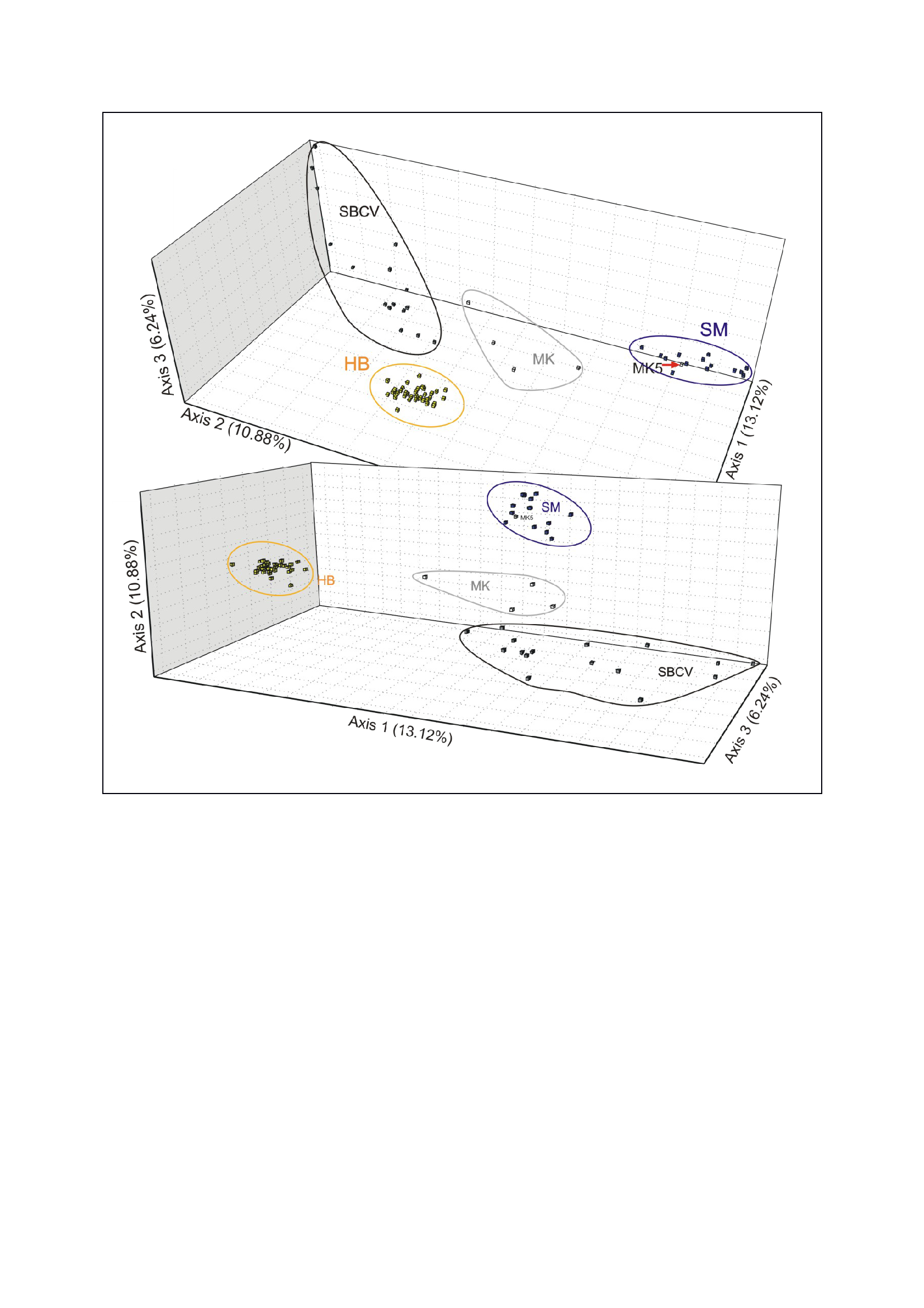

Supplement: S4 Fig — (TIFF) [file pone.0117003.s004.tiff]

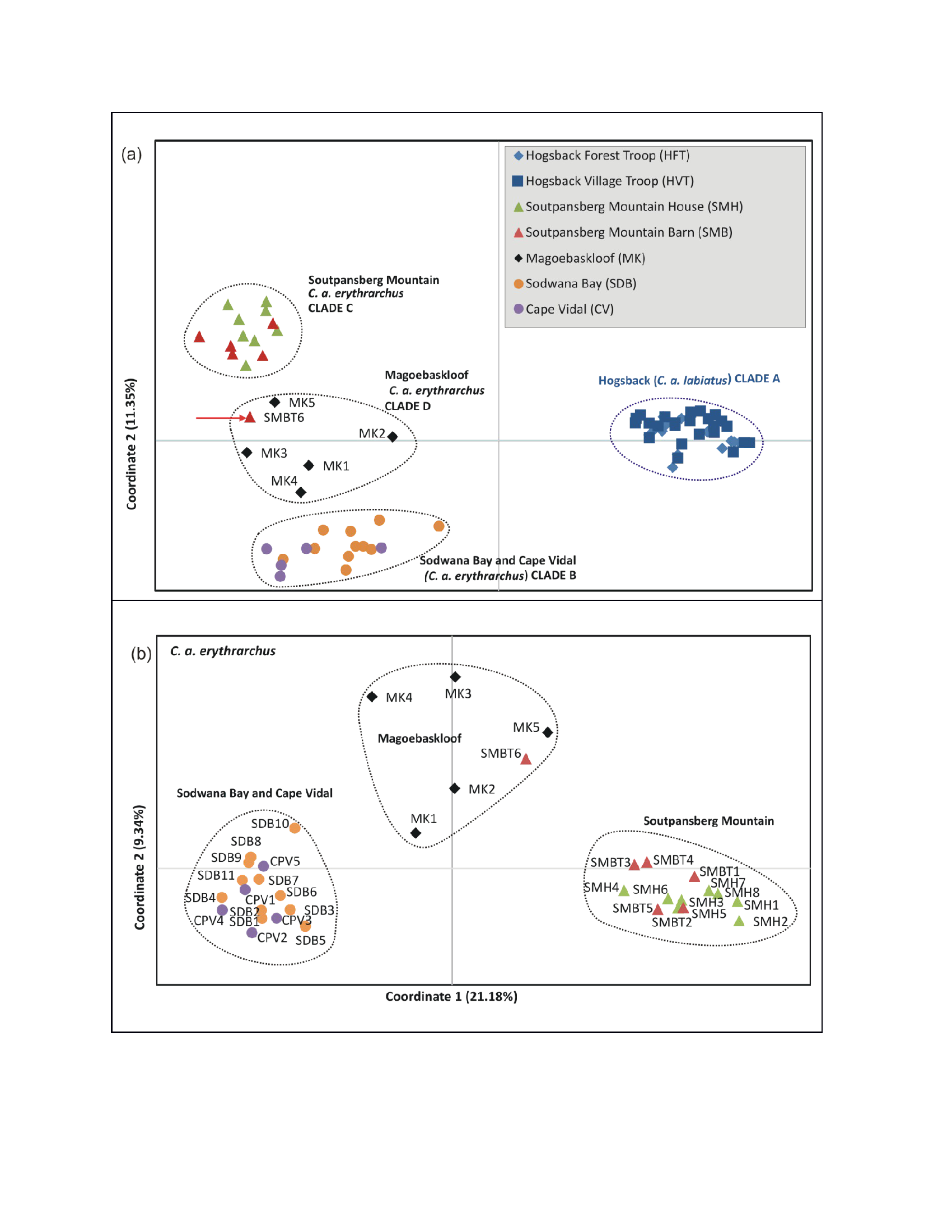

Supplement: S5 Fig — (a) and (b) are different projections of the same results. Individuals are colour-coded according to their collection locality (HB, Hogsback; SBCV, Sodwana Bay and Cape Vidal; MK, Magoebaskloof; and SM, Soutpansberg Mountains). (TIFF) [file pone.0117003.s005.tiff]

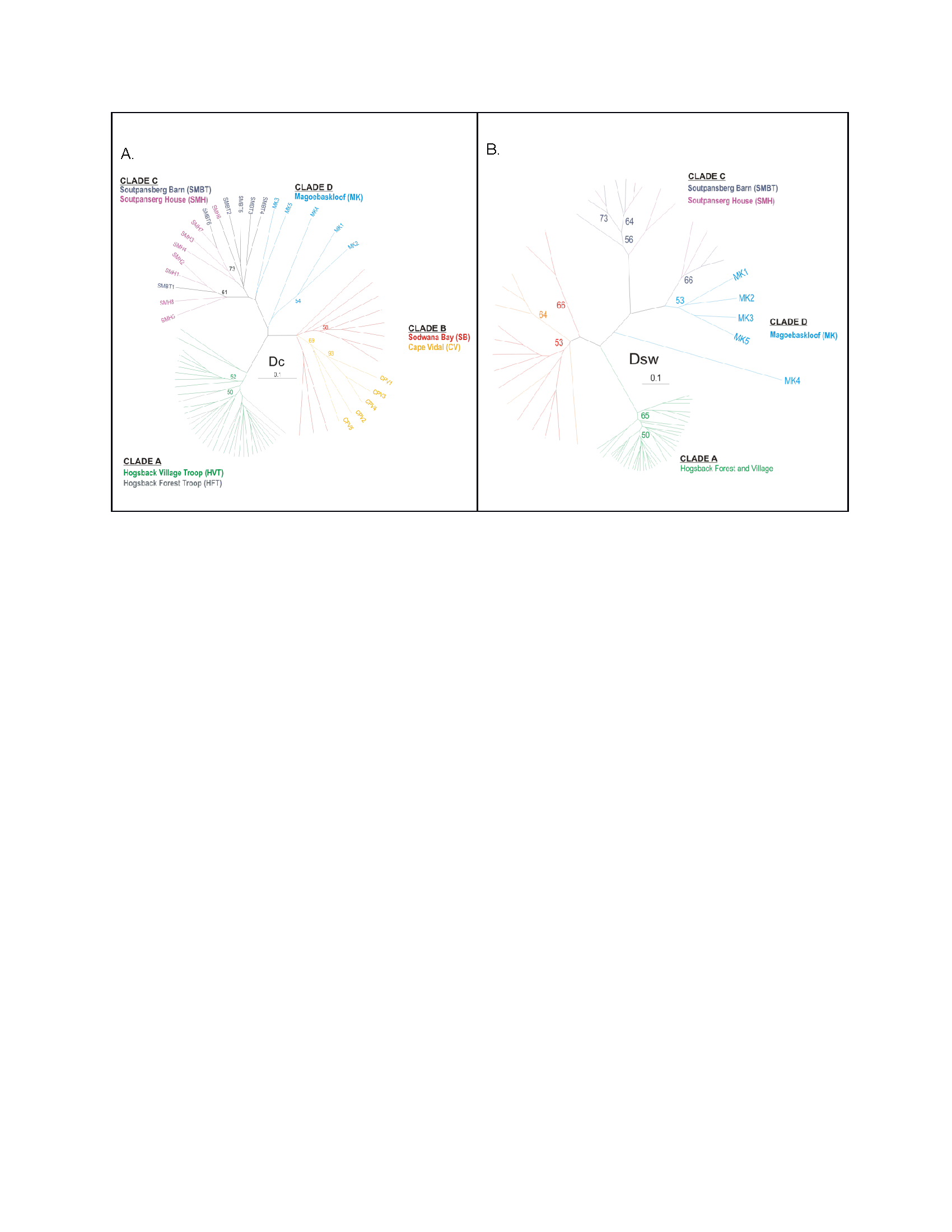

Supplement: S6 Fig — (TIFF) [file pone.0117003.s006.tiff]

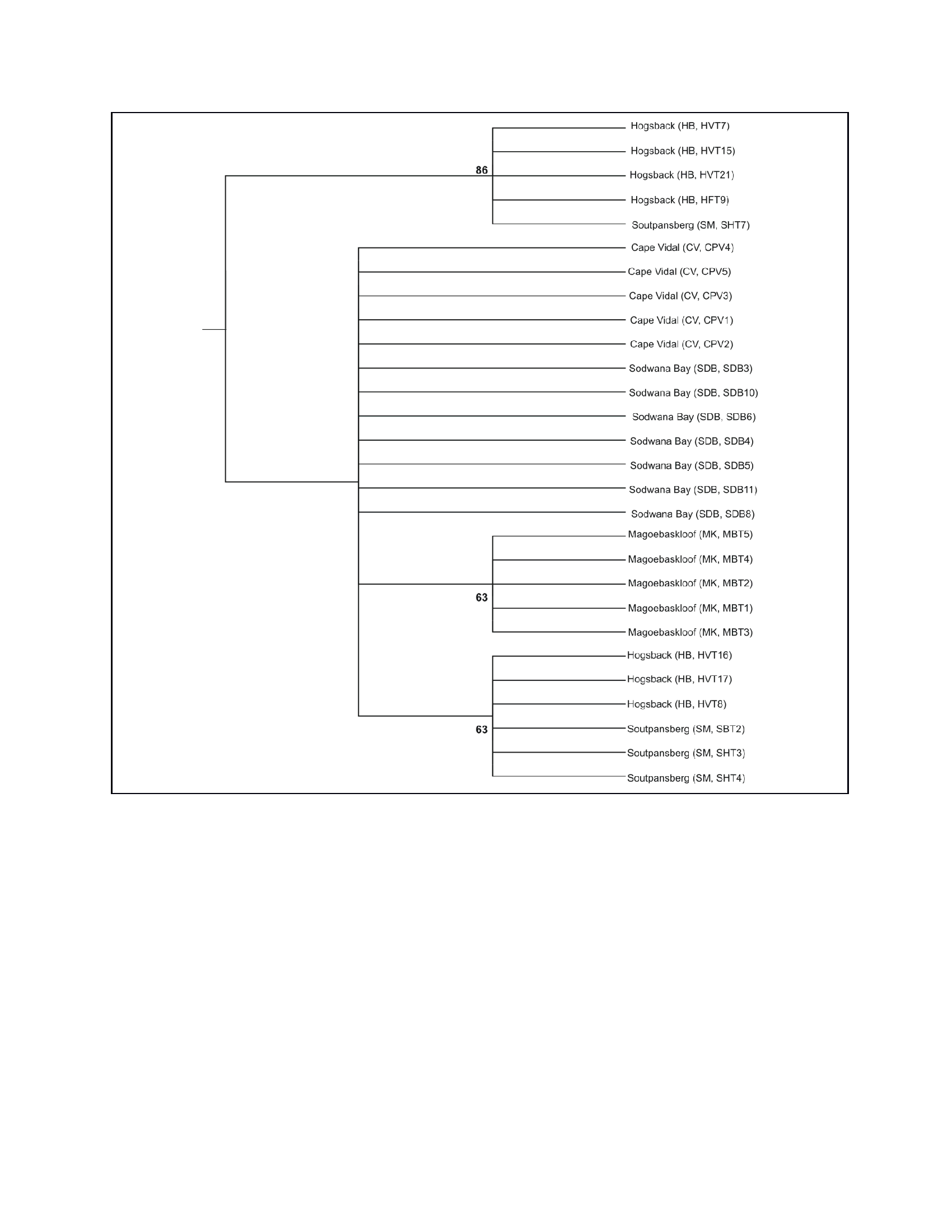

Supplement: S7 Fig — Numbers at the nodes are bootstrap support. (TIFF) [file pone.0117003.s007.tiff]

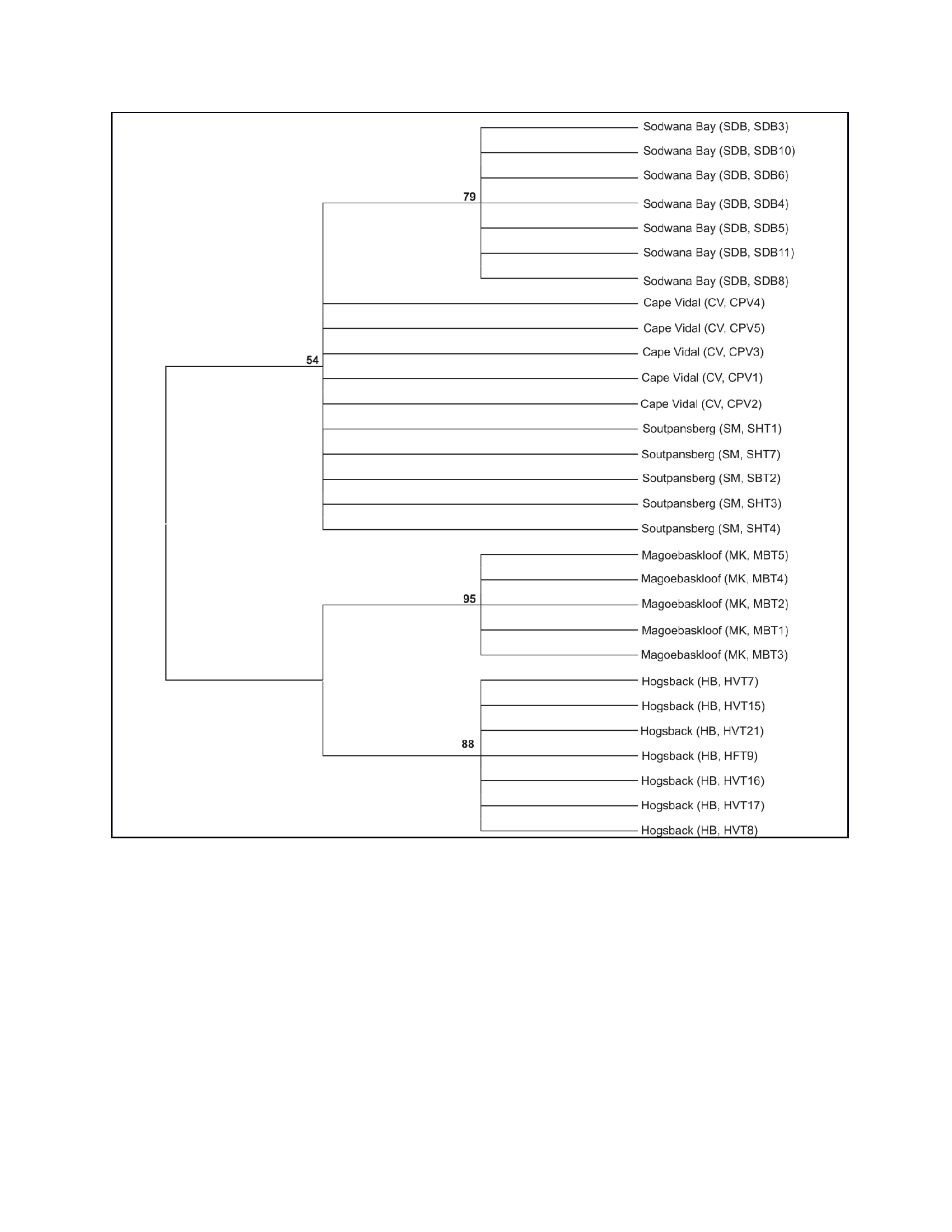

Supplement: S8 Fig — Numbers at the nodes are bootstrap support. (TIFF) [file pone.0117003.s008.tiff]

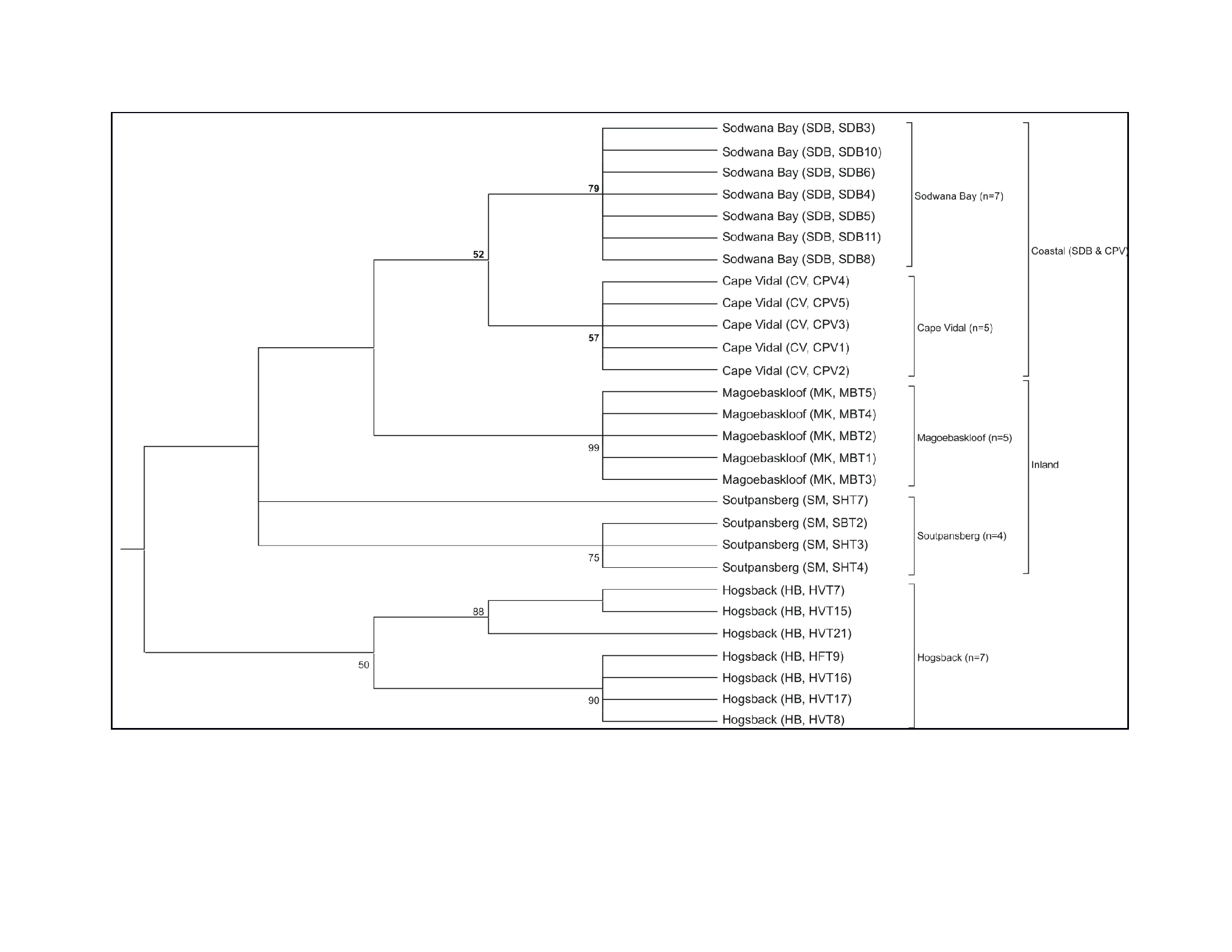

Supplement: S9 Fig — Numbers at the nodes are bootstrap support (<50% not shown). (TIFF) [file pone.0117003.s009.tiff]

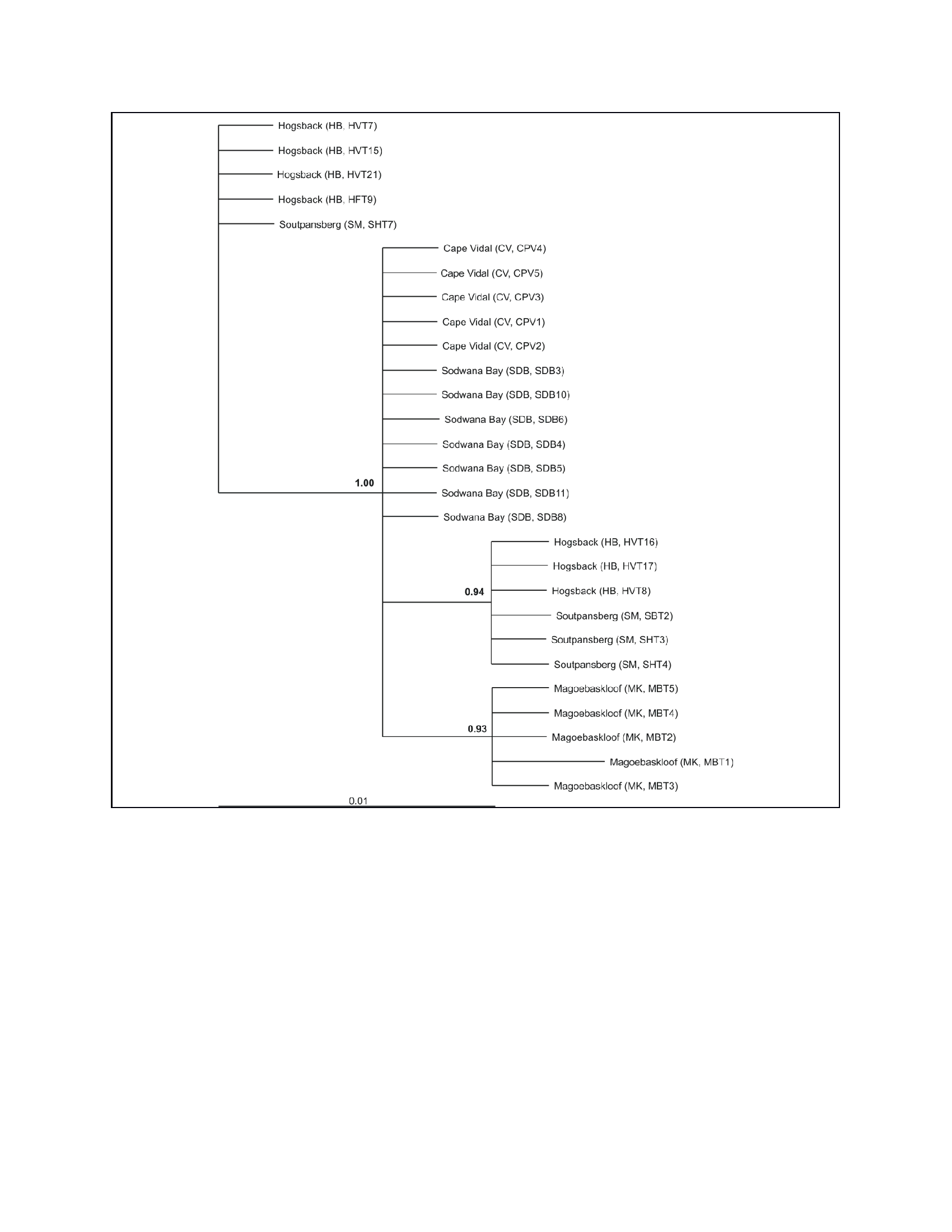

Supplement: S10 Fig — Numbers at the nodes are Bayesian posterior probabilities. (TIFF) [file pone.0117003.s010.tiff]

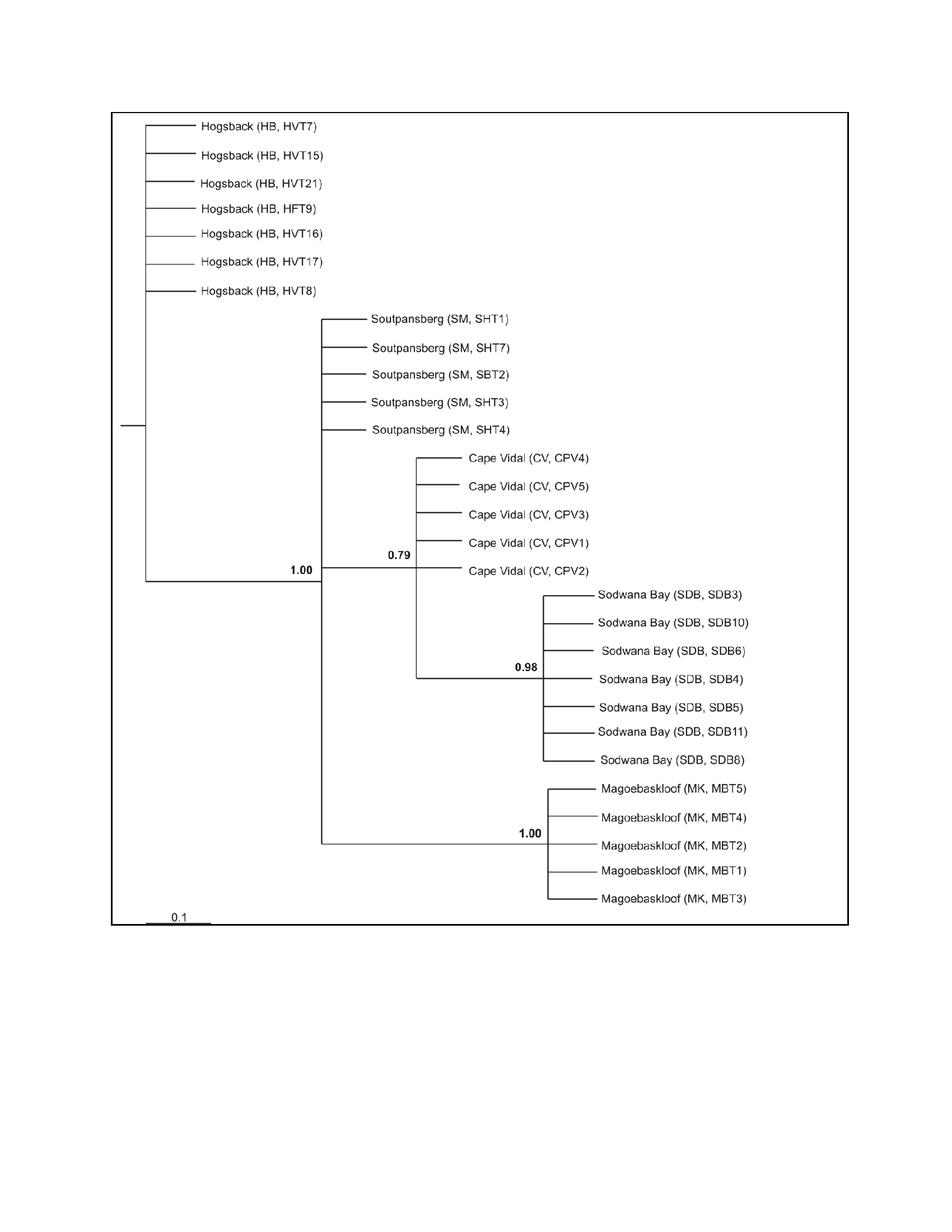

Supplement: S11 Fig — Numbers at the nodes are posterior probabilities. (TIFF) [file pone.0117003.s011.tiff]

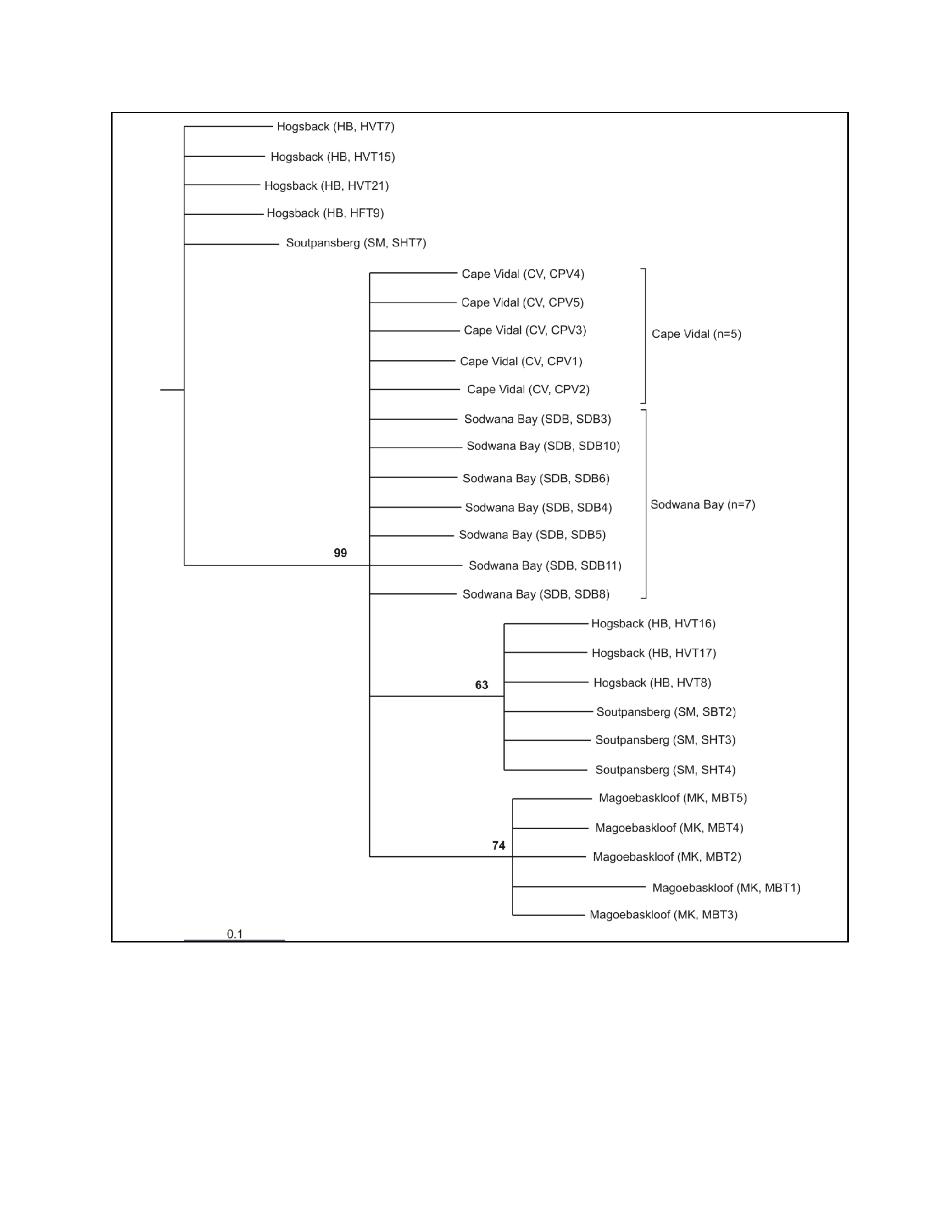

Supplement: S12 Fig — Numbers at the nodes are posterior probabilities. (TIFF) [file pone.0117003.s012.tiff]

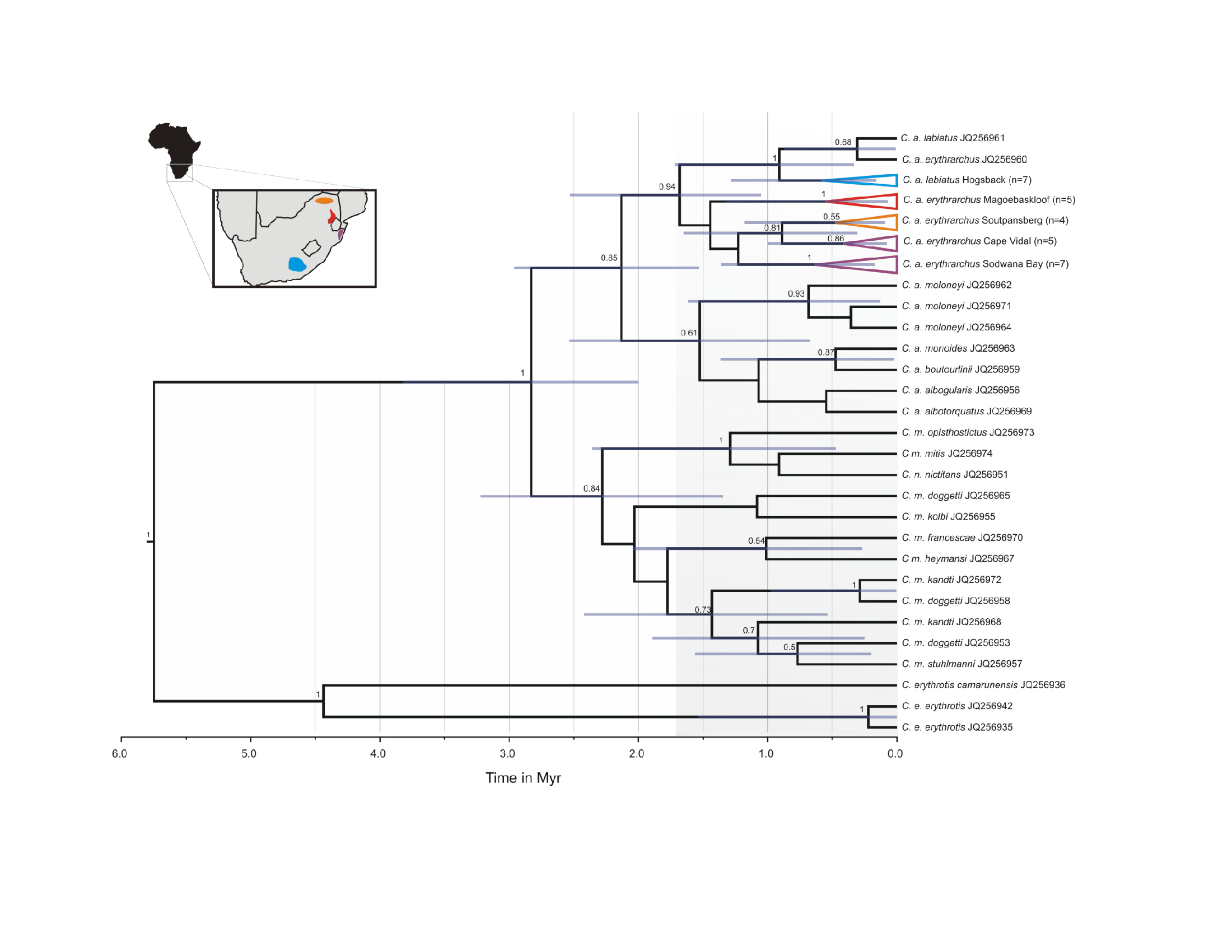

Supplement: S13 Fig — (TIFF) [file pone.0117003.s013.tiff]
