# Supplementary material for: New Insights into Samango Monkey Speciation in South Africa
Source: PLoS One. 2015 Mar 23;10(3):e0117003. doi: 10.1371/journal.pone.0117003 (PMC4370472; doi:10.1371/journal.pone.0117003)
Supplement: S1 Table — (DOCX) [file pone.0117003.s014.docx]

| **Locality** | **Month** | **Number females** | **Number males** | **Total individuals** | **Total hairs** |
| --- | --- | --- | --- | --- | --- |
| Soutpansberg | August | 6 | 2 | 8 | 40 |
| Magoebaskloof | August | 3 | / | 3 | 15 |
| Sodwana Bay | May | 3 | 2 | 5 | 25 |
| Cape Vidal | May | / | 3 | 3 | 15 |
| Hogsback | March, August, September | 8 | 7 | 15 | 75 |
