# Supplementary material for: New Insights into Samango Monkey Speciation in South Africa
Source: PLoS One. 2015 Mar 23;10(3):e0117003. doi: 10.1371/journal.pone.0117003 (PMC4370472; doi:10.1371/journal.pone.0117003)
Supplement: S2 Table — (DOCX) [file pone.0117003.s015.docx]

| **Male** |  |  |  |  |  |  |  |
| --- | --- | --- | --- | --- | --- | --- | --- |
| **Variable** | **Axis 1** | **Axis 2** | **Axis 3** | **Axis 4** | **Axis 5** | **Axis 6** | **Axis 7** |
| **BM (kg)** | 0.75 | -0.05 | -0.29 | -0.52 | 0.29 | 0.00 | 0.02 |
| **NC** | 0.30 | -0.65 | 0.22 | 0.46 | 0.18 | -0.39 | -0.17 |
| **HB** | 0.22 | -0.20 | 0.49 | -0.09 | -0.28 | 0.68 | -0.34 |
| **TL** | 0.41 | 0.51 | -0.26 | 0.51 | -0.29 | -0.05 | -0.39 |
| **HF** | 0.32 | 0.15 | 0.22 | 0.37 | 0.05 | 0.24 | 0.79 |
| **EL** | 0.12 | -0.27 | -0.12 | -0.20 | -0.85 | -0.27 | 0.26 |
| **EW** | -0.12 | -0.42 | -0.71 | 0.26 | 0.01 | 0.49 | 0.05 |
| **Female** |  |  |  |  |  |  |  |
| **BM (kg)** | 0.64 | -0.19 | 0.66 | -0.06 | 0.14 | -0.32 | -0.02 |
| **NC** | 0.06 | -0.89 | -0.37 | -0.08 | 0.24 | 0.01 | 0.06 |
| **HB** | 0.19 | -0.09 | 0.22 | 0.24 | -0.13 | 0.76 | 0.50 |
| **TL** | 0.70 | 0.27 | -0.62 | 0.03 | -0.13 | -0.11 | 0.17 |
| **HF** | 0.23 | -0.09 | -0.03 | 0.25 | -0.14 | 0.39 | -0.84 |
| **EL** | -0.10 | -0.08 | -0.01 | 0.93 | 0.01 | -0.34 | 0.09 |
| **EW** | -0.05 | -0.28 | 0.06 | -0.09 | -0.93 | -0.20 | 0.05 |
