# Supplementary material for: New Insights into Samango Monkey Speciation in South Africa
Source: PLoS One. 2015 Mar 23;10(3):e0117003. doi: 10.1371/journal.pone.0117003 (PMC4370472; doi:10.1371/journal.pone.0117003)
Supplement: S3 Table — (DOCX) [file pone.0117003.s016.docx]

| **Locus pair** |  | **P-Value** |
| --- | --- | --- |
| a | e | 0.001 |
| d | u | 0.014 |
| c | g | 0.018 |
| g | i | 0.028 |
| b | p | 0.030 |
| i | r | 0.031 |
